# Aberrant one-carbon metabolism and ancestral genetics underlie edematous severe acute malnutrition

**DOI:** 10.1038/s41467-026-74239-x

**Published:** 2026-10-15

**Authors:** Natasha C. Lie, Yixing Han, Qing Li, Aarti Jajoo, Jared Redmond, Aparna Haldipur, Solome Zewdu, Emilyn Banfield, Shanker Swaminathan, Sharon Howell, Orgen Brown, Roa Sadat, Nancy J. Hall, Kwesi Marshall, Katharina V. Schulze, Thaddaeus May, Marvin E. Reid, Carolyn Taylor-Bryan, Mark J. Manary, Indi Trehan, Mamana Mbiyavanga, Wisdom A. Akurugu, Colin A. McKenzie, Dhriti Sengupta, Elizabeth G. Atkinson, Ananyo Choudhury, Neil A. Hanchard

**Affiliations:** 1 Graduate Program in Integrative Molecular and Biomedical Sciences, Baylor College of Medicine, Houston, TX USA; 2 Childhood Complex Disease Genomics Section, Center for Precision Health Research, National Human Genome Research Institute, National Institutes of Health, Bethesda, MD USA; 3 Department of Molecular and Human Genetics, Baylor College of Medicine, Houston, TX USA; 4 Tropical Metabolism Research Unit, Caribbean Institute for Health Research, University of the West Indies, Mona, Jamaica; 5 International Centre for Diarrhoeal Disease Research, Dhaka, Bangladesh; 6 Department of Internal Medicine, Baylor College of Medicine, Houston, TX USA; 7 USDA/ARS/Children’s Nutrition Research Center, Baylor College of Medicine, Houston, TX USA; 8 Department of Community Health, Kamuzu University of Health Sciences, Blantyre, Malawi; 9 Department of Pediatrics, Washington University in St. Louis, St. Louis, MO USA; 10 Department of Pediatrics, University of Washington, Seattle, WA USA; 11 Department of Global Health, University of Washington, Seattle, WA USA; 12 Department of Epidemiology, University of Washington, Seattle, WA USA; 13 Computational Biology Group, Faculty of Health Sciences, University of Cape Town, Cape Town, Western Cape South Africa; 14 Sydney Brenner Institute for Molecular Bioscience (SBIMB), University of the Witwatersrand, Johannesburg, South Africa; 15 Jan and Dan Duncan Neurological Research Institute, Texas Children’s Hospital, Houston, TX USA

**Keywords:** Genetics research, Malnutrition, Medical genomics

## Abstract

Edematous severe childhood malnutrition is distinct from the non-edematous form of malnutrition and remains a major cause of under-five mortality. Its etiology remains unclear, although aberrant one-carbon metabolism has emerged as a potential contributor. Here, we assess genetic variants at loci known to influence one-carbon metabolism, and determine their association with edematous malnutrition in Jamaica and Malawi. Six loci show evidence of association, including four related to homocysteine and folate metabolism - *AHCYL1*, *PRICKLE2*, *GABBR2*, and *PLD2*, with SNPs at the latter three also having direct effects on homocysteine-related metabolites in Mendelian Randomization. Collectively, one-carbon metabolism-related variants were significantly associated with edematous malnutrition (z = 3.2), with population differences linked to a shared East African genetic ancestry and distinct signatures of selection. These findings strengthen evidence implicating aberrant one-carbon metabolism in edematous malnutrition and highlight the utility of cell-free DNA, intra-continental admixture, and population genetics for mapping disease risk.

## Abstract (web summary)

Edematous malnutrition in children has unclear causes. Here, the authors show that genetic variation at one‑carbon metabolism genes influences disease risk through homocysteine‑related pathways and ancestry‑linked effects in affected populations.

## Introduction

Severe acute malnutrition (SAM) affects 16.9 million children worldwide, predominantly those under five years old, and either directly or indirectly contributes to more than one million childhood deaths per year^1,2^. SAM is clinically characterized by a weight-for-height that is more than three standard deviations below the median, a mid-upper-arm circumference of less than 115 mm, or the presence of nutritional edema^3^. In practice, SAM is recognized to occur in two clinically distinct forms: edematous SAM (ESAM), which includes the syndromes of kwashiorkor and marasmic-kwashiorkor, and non-edematous SAM (NESAM), also known as marasmus^4^. ESAM accounts for a higher proportion of hospitalized SAM in east- and central- Africa and the Caribbean^5,6^, and is associated with mortality rates of 10–20% among those who are hospitalized with SAM and medical complications^7–9^. ESAM is clinically characterized by bilateral pitting edema (swelling) of the extremities and severe systemic involvement that may include skin and hair changes, fatty liver, and multi-system organ failure^4,10,11^. By contrast, NESAM is more prevalent in Southeast Asia and northern- and western African geographies, and is characterized by generalized wasting of body tissues, typically with less severe systemic involvement^4,6^. Whilst it is well-recognized that the development of SAM is largely linked to food security and availability, the persistence of adverse geopolitical factors mean that SAM is still highly prevalent globally.

ESAM was first described in medical literature in the early part of the 20th century^12–14^; however, the reasons why a severely malnourished child will develop ESAM as opposed to NESAM remain largely unknown^15^. In the past, it was hypothesized that diet or protein intake were primary contributors to ESAM risk; however, decades of epidemiological surveys have failed to show consistent evidence for differences in protein intake or any other single dietary deprivation between children who develop ESAM and those who develop NESAM^16–19^. Similarly, there have been no consistently demonstrated differences in infectious or environmental exposures between children who develop ESAM and those who develop NESAM^4,20–22^. There are, however, numerous pathophysiological differences between the two clinical manifestations, particularly in their metabolism and biochemistry; for example, ESAM has been associated with a slower rate of protein breakdown in starvation, impaired heparin sulfate proteoglycan expression, greater dietary cysteine efficiency, and increased oxidative stress, compared to children with NESAM^23–28^. Despite this, it remains unknown whether these observations represent downstream effects that result from the disorder or are primary etiological differences. Perhaps as a result, ESAM and NESAM are still treated in the same manner, using the same nutritional supplements, despite well described differences in pathophysiology and underlying biochemistry.

Among biochemical differences between children with ESAM and those with NESAM, the cellular movement of methyl groups known as one-carbon metabolism (OCM) has been a particular recent focus. OCM plays a major role in health and disease, providing substrates for mitochondrial activity and ATP generation, deoxynucleoside triphosphates (dNTPs) for DNA repair, and methyl groups needed for the maintenance of DNA and other cellular methylation events in mitotically active cells^29^. The broad metabolic reach of OCM has been hypothesized as a potential unifying factor for the multisystem dysfunction seen in ESAM, and recent studies lend support to this. For instance, metabolomic studies have demonstrated that children with ESAM have significantly lower serum levels of the essential amino acid methionine^30^ with concomitant related effects, including dysregulation of asymmetric dimethyl arginine (ADMA), lower cysteine levels, impaired cysteine synthesis, reduced acylcarnitine production, and impaired fatty acid transport^23,25^. Mice fed a maize-vegetable diet similar to that seen among SAM children in Malawi develop a fatty liver phenotype that is similar to that seen in ESAM, and is abrogated by supplementation of the methyl-group donor choline^31^. Similarly, although there are no overt differences in microbiome composition between NESAM and ESAM children, a metagenomic study of severe malnutrition, conducted without regard to subtype, reported significantly lower OCM metabolite levels among gnotobiotic mice transplanted with stool from children with ESAM^32^.

Observations of OCM dysfunction in ESAM are also consistent with stable isotope whole-body flux studies reporting slower methionine flux among children with ESAM^26^. We have previously shown that during the acute nutritional stress, children with ESAM have significantly lower buccal cell DNA methylation than children with NESAM^33^, and this was consistent with observations of methionine turnover. Notably, and consistent with turnover studies conducted in children who had recovered from SAM, methylation differences were not observed among adults who had recovered from having either ESAM or NESAM as children, confirming that OCM dysfunction is a feature of the acute nutritional insult. Significantly hypomethylated loci were associated with genes both directly and indirectly implicated in the ESAM phenotype, and with loci associated with disordered nutrition states (e.g., obesity) and body anthropometry. Thus, collectively, there is a growing body of evidence supporting OCM dysfunction in ESAM. These findings, however, do not directly explain why only some children have the OCM derangements associated with ESAM, whilst others from the same region and environment have the OCM profile associated with NESAM.

Although there are no formal heritability estimates for ESAM, children with repeated bouts of SAM are more likely to develop the same form of SAM (i.e., either ESAM or NESAM) upon readmission to hospital^34,35^; this observation, alongside the well-documented lack of demonstrable environmental, infectious, or metagenomic differences between ESAM and NESAM, has focused attention on the potential genetic risk of ESAM, which is consistent with recent interest in the genetics of malnutrition^36^. To date, however, attempts at genetic association have been limited to three single-candidate SNP association studies conducted in sample sizes of less than 200 individuals decades ago^37–39^. We thus sought to take a modern genetic approach to understanding the risk of developing ESAM, leveraging advances that have characterized genetic risk association studies of other public health disorders, including nutritional disorders like obesity. Such an approach has the potential to identify novel biological pathways or confirm the role for specific metabolic pathways in the development of ESAM. These pathways can then serve as targets for therapeutic and/or nutritional interventions. OCM is a fundamental metabolic pathway that has been genetically characterized^40,41^, with well-curated genes and loci mediating interindividual metabolic variation. Therefore, we started by evaluating the potential impact of genetic variation at OCM loci on the risk of developing ESAM.

**Fig. 1:**
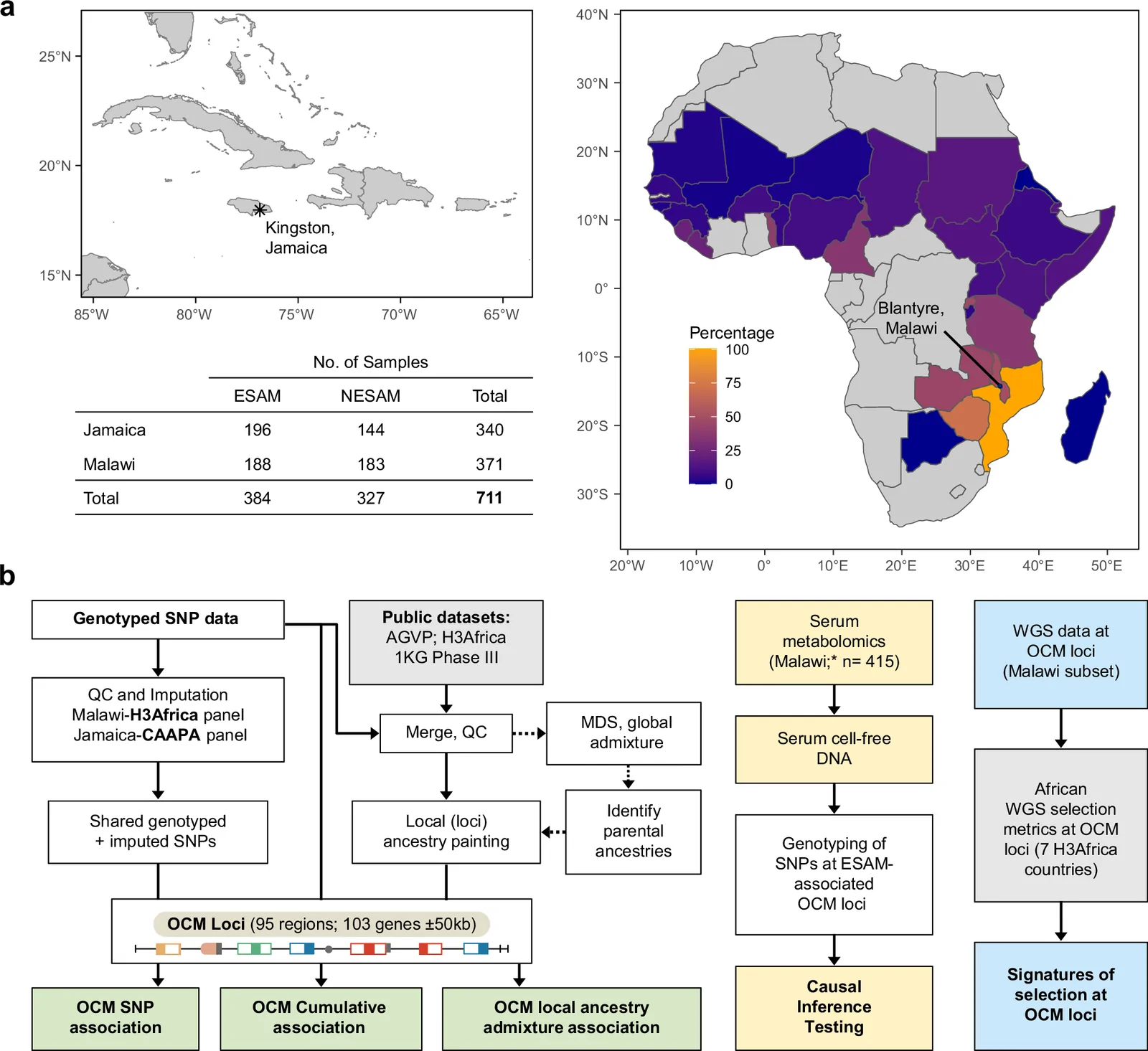
Study design and samples. **a** SAM samples and study recruitment in Jamaica and Malawi are shown alongside the prevalence (%) of nutritional edema (ESAM) across Africa from “Putting Kwashiorkor on the Map”^6^. **b** Overview of major study analyses and their contributing datasets; association studies are shown in green, with adjunctive studies in orange and blue. Gray boxes represent external (publicly available) datasets. *study of serum OCM metabolites^30^. Maps generated using ggplot2^90^ in R^76^.

In this study, we compile a comprehensive list of OCM-associated genetic loci and evaluate genetic association with ESAM at these loci in population samples from Jamaica and Malawi. We then leverage cell free DNA and causal inference testing; whole genome sequencing and signatures of selection; as well as local and global population ancestry, to identify, verify, and clarify OCM-genetic loci associated with ESAM (Fig. 1).

## Results

Our primary analyses utilized DNA samples from 833 individuals from Jamaica and Malawi approximately evenly split between ESAM (case/affected group) and NESAM (reference/control group) at each location (Fig. 1; Supplementary Data 1). Samples were genotyped on the Illumina H3Africa array, with subsequent quality control (QC) processing resulting in 711 samples and 1,639,325 genotyped SNPs (“Methods”). Among genotyped SNPs, 9479 SNPs fell within 50 kb (±) of a curated list of 103 autosomal genes and loci previously associated with OCM (“Methods”; Supplementary Data 2); this included four gene clusters with two or more genes in tandem. After variant imputation and quality control (“Methods”), a set of 45,411 imputed and 9479 genotyped SNPs at 95 OCM loci were identified to test for association and perform downstream analyses (Supplementary Fig. 1).

### Variation at OCM loci is associated with ESAM

**Fig. 2:**
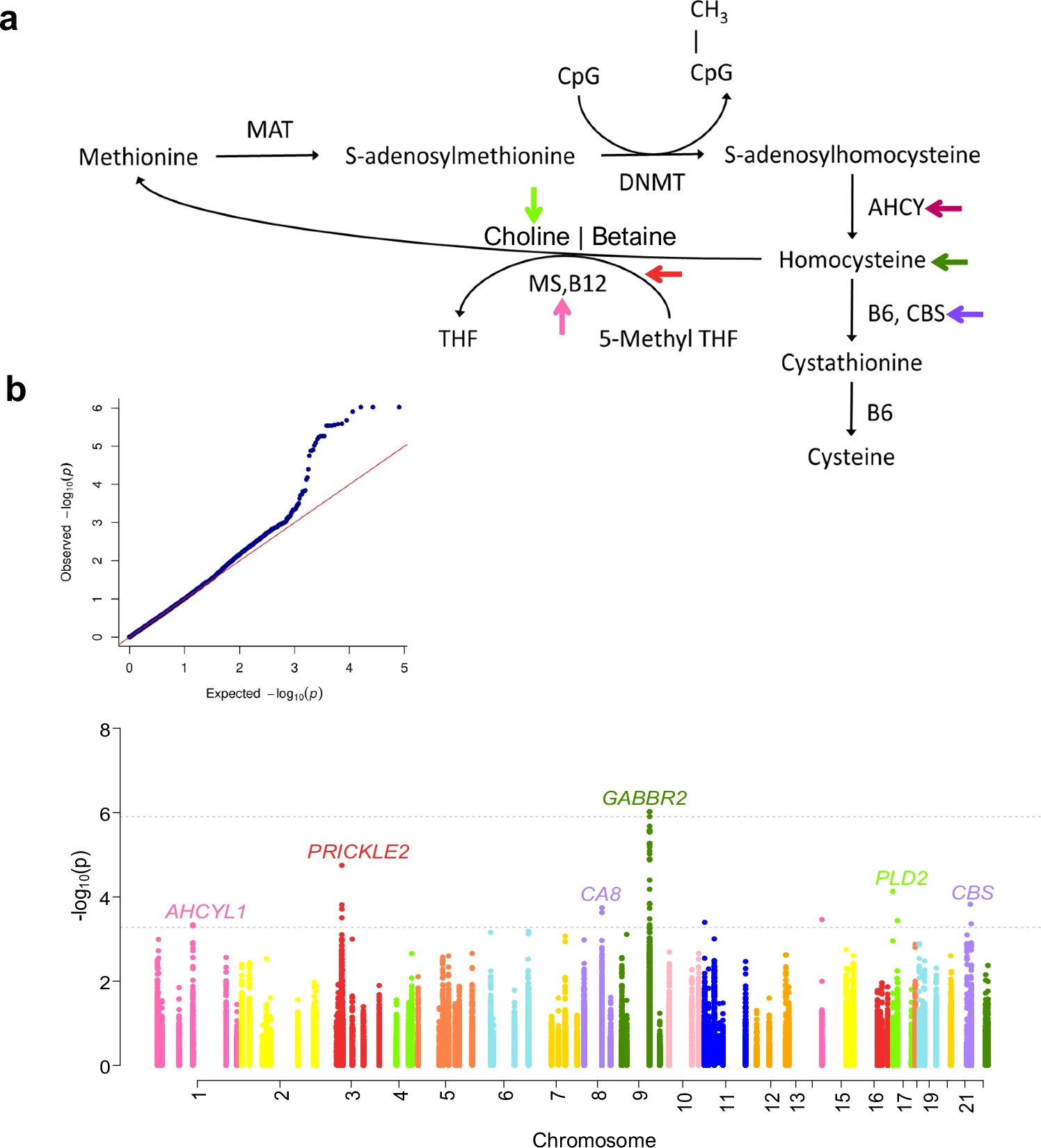
One carbon metabolism and genetic association with ESAM. **a** The one-carbon metabolism (OCM) cycle showing the folate and methionine cycles. In the folate cycle, dietary folate is processed to 5-methyl-tetrahydrofolate, which is then used in the production of methionine from homocysteine. Methionine is processed in its eponymous cycle to produce homocysteine, which contributes methyl groups for DNA- and other cellular component methylation and can be remethylated to methionine through the folate cycle. MAT Methionine adenosyltransferase, DNMT DNA methyltransferase, AHCY Adenosylhomocysteinase, THF Tetrahydrofolate; 5-Methyl THF – 5-methyltetrahydrofolate, MS Methionine synthase, B6 Vitamin B6, B12 Vitamin B12, CBS Cystathionine β-synthase. **b** QQ plot and Manhattan plot of SNP (genotyped and imputed) association with ESAM demonstrating candidate loci. Association testing was performed using a linear mixed model implemented in GEMMA (Wald test). All tests were two-sided. The activity of loci reaching the locus threshold for significance (dashed line at –log_10_P= 3.27) or the SNP threshold for significance (dashed line at –log_10_P= 5.9) in OCM is represented by colored arrows in 2a matching the locus colors in 2b. Significance thresholds are adjusted for multiple comparisons via Bonferroni correction.

We first assessed the association between imputed SNPs at OCM loci and ESAM using the linear mixed model implemented in GEMMA (see “Methods”). A total of 56 SNPs at nine OCM loci, surpassed a secondary significance threshold adjusted for the number of loci (*n* = 95) tested (*p* < 5.26 × 10^−4^) (Fig. 2). This latter threshold was supported by an excess of observed *p* values without evidence of test-wide inflation (λ = 1.02) on the resulting Quantile-Quantile (QQ) plot (Fig. 2). To further reduce potential false positives, we characterized candidate loci as those with multiple SNPs surpassing our locus-level cut-off or single variants with evidence of association using a secondary method (PLINK; *n* = 2 loci). This resulted in a final set of six ESAM-associated OCM loci (Supplementary Data 3).

The strongest associated locus was an intragenic region on chromosome 9q22.33 (Supplementary Fig. 2a) falling within the first intron of gamma-aminobutyric acid type B receptor subunit two (*GABBR2*; top SNP rs7038285, two-sided *p* = 6.98×10^−7^, Odds Ratio (OR) = 0.85), where the minor allele (C) was enriched among individuals with NESAM. SNPs within intron 13 of *GABBR2* ( ~ 260 kb upstream of our top SNPs) have been associated with lower plasma homocysteine^40^ in a European population. The mechanistic relationship of this association to the *GABBR2* gene, which is a member of the G-protein coupled receptor 3- and GABA-B receptor families, are unclear, however. We also found similar evidence of putative association across an intronic region in *PRICKLE2* on chromosome 3p14.1 (top SNP rs11130959, two-sided *p *= 4.11 × 10^−5^, OR = 0.88) (Supplementary Fig. 2b), with the minor allele also being enriched among NESAM participants. The canonical function of *PRICKLE2* is unknown, although it has been associated with variation in folate levels^42^. Of the six top candidate loci, four (*AHCYL1*, *PRICKLE2*, *GABBR2*, and *PLD2*) directly impact either conversion to- or breakdown of- homocysteine (Fig. 2), including a coding variant - rs1052748 (p.Thr577Ile)—in exon 17 of *PLD2* that was enriched among ESAM individuals (Supplementary Fig. 2c)*. PLD2* encodes phospholipase D2, which generates the methyl-group donor choline through the hydrolysis of phosphatidylcholine. The rs1052748 variant is reported as an expression quantitative trait locus (eQTL) for *PLD2* in multiple tissues in the Genotype-Tissue Expression (GTEx) Portal^43^.

**Fig. 3:**
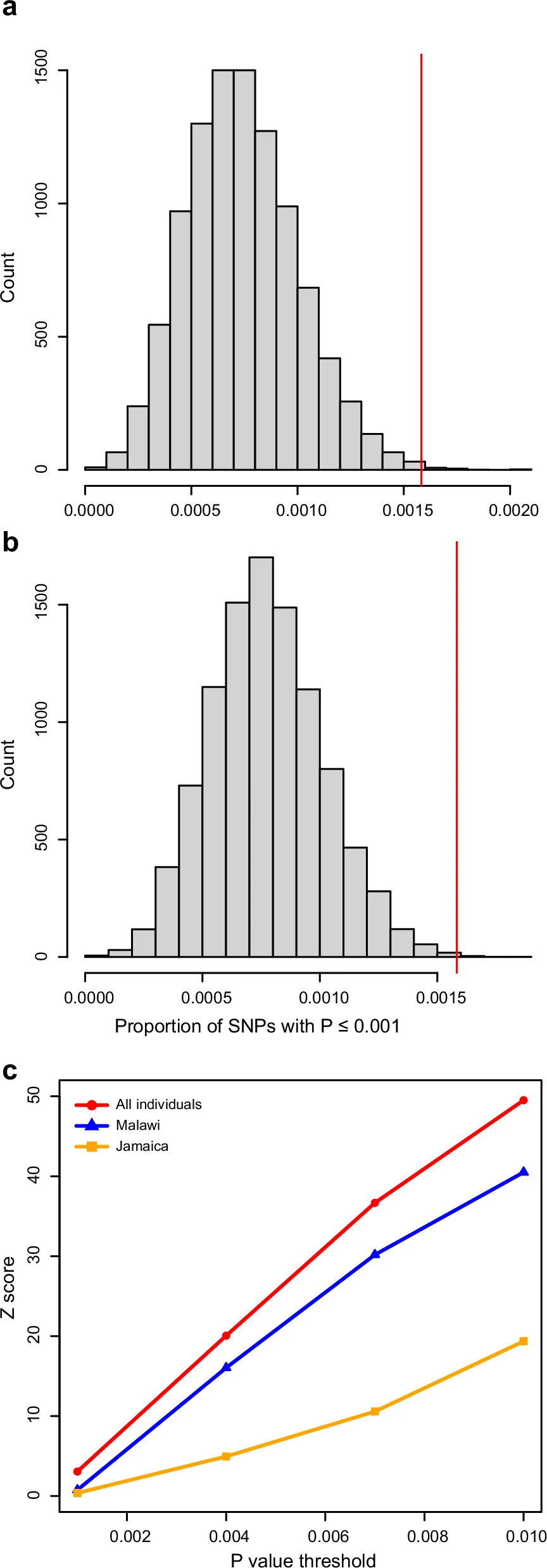
Cumulative association of OCM SNPs. Each panel shows a histogram illustrating the proportion of 9479 randomly sampled SNPs surpassing the significance threshold of p < 0.001, compared to the number of SNPs in OCM loci passing the same threshold (red line). All tests were one-sided. Statistical significance was assessed using an empirical permutation test; p values were calculated as the proportion of permutations exceeding the observed value. No multiple-comparison adjustment was applied. **a** SNPs randomly sampled from the total genotyped pool. **b** SNPs randomly sampled from a pool of ketone metabolism loci. **c** Z scores of random sampling experiments for *p* value thresholds between locus-level significance threshold and a *p* value ceiling of *p* = 0.01 (see “Methods”).

Metabolic flux results from cumulative enzymatic activity at multiple points within a biochemical cycle. Given our observation of multiple putatively associated OCM SNPs, each with variable effects on OCM, we considered the potential for a cumulative effect of OCM-associated SNPs on ESAM susceptibility. To test this, we applied a permutation test (“Methods”) to the association statistics of OCM SNPs and compared this with the association tests of the ~1.8 million genotyped SNPs genome wide. At a *p* value cut-off of <0.001, a greater proportion of SNPs at OCM loci were associated with ESAM relative to the randomly sampled dataset (z = 3.2; Fig. 3a). At more permissive *p* value thresholds, the z-score differentiating our OCM SNPs and the random sampling experiment was even more pronounced (e.g., at a threshold of *p* = 0.01, the resulting z-score was 50 (Fig. 3c)).

Next, we considered whether our cumulative association might have resulted from comparing SNPs in linkage disequilibrium (LD) at metabolically related loci to a random distribution of largely unlinked SNPs. To do so, we repeated the permutation test, but this time randomly sampling the same number of SNPs (*n* = 9479) from the 51,105 SNPs found at loci related to ketone metabolism, which is not known to be associated with SAM (“Methods”). Relative to this ketone metabolism background, the proportion of OCM SNPs showing association was still in the upper tail of the distribution (z = 3.19) at the *p* < 0.001 threshold (Fig. 3b). Finally, as a negative control, we performed the same genome-wide comparison as above for SNPs at sphingolipid metabolism loci, which has a similar number of SNPs to OCM, but no a priori evidence for involvement in ESAM. The proportion of sphingolipid metabolism SNPs showing association with ESAM fell well within the randomly generated distribution (z = −0.70).

There are few established ex vivo models for evaluating genetic variation in SAM. Therefore, we sought to provide support for the proposed functional impact of associated variants by assessing the impact of ESAM-associated SNPs on OCM metabolites in the context of ESAM vs NESAM. We first extracted cell-free DNA (cfDNA) from ~400 serum samples (“Methods”) from our previous study of OCM metabolites in ESAM/NESAM, which was done in an independent cohort of children from Malawi recruited around the time of their SAM diagnosis^30^. We then genotyped four ESAM-associated SNPs rs753562 (*PRICKLE2*), rs17664202 (*PRICKLE2)*, rs7869446 (*GABBR2*), and rs1052748 (*PLD2*) using cfDNA samples (Supplementary Information; Supplementary Data 4). Three SNPs (rs17664202 was out of Hardy-Weinberg equilibrium) were then used as instrumental variables in a two-sample Mendelian Randomization (MR) to assess the contributing effect of each of the 16 OCM metabolites on ESAM risk (“Methods”).

**Fig. 4:**
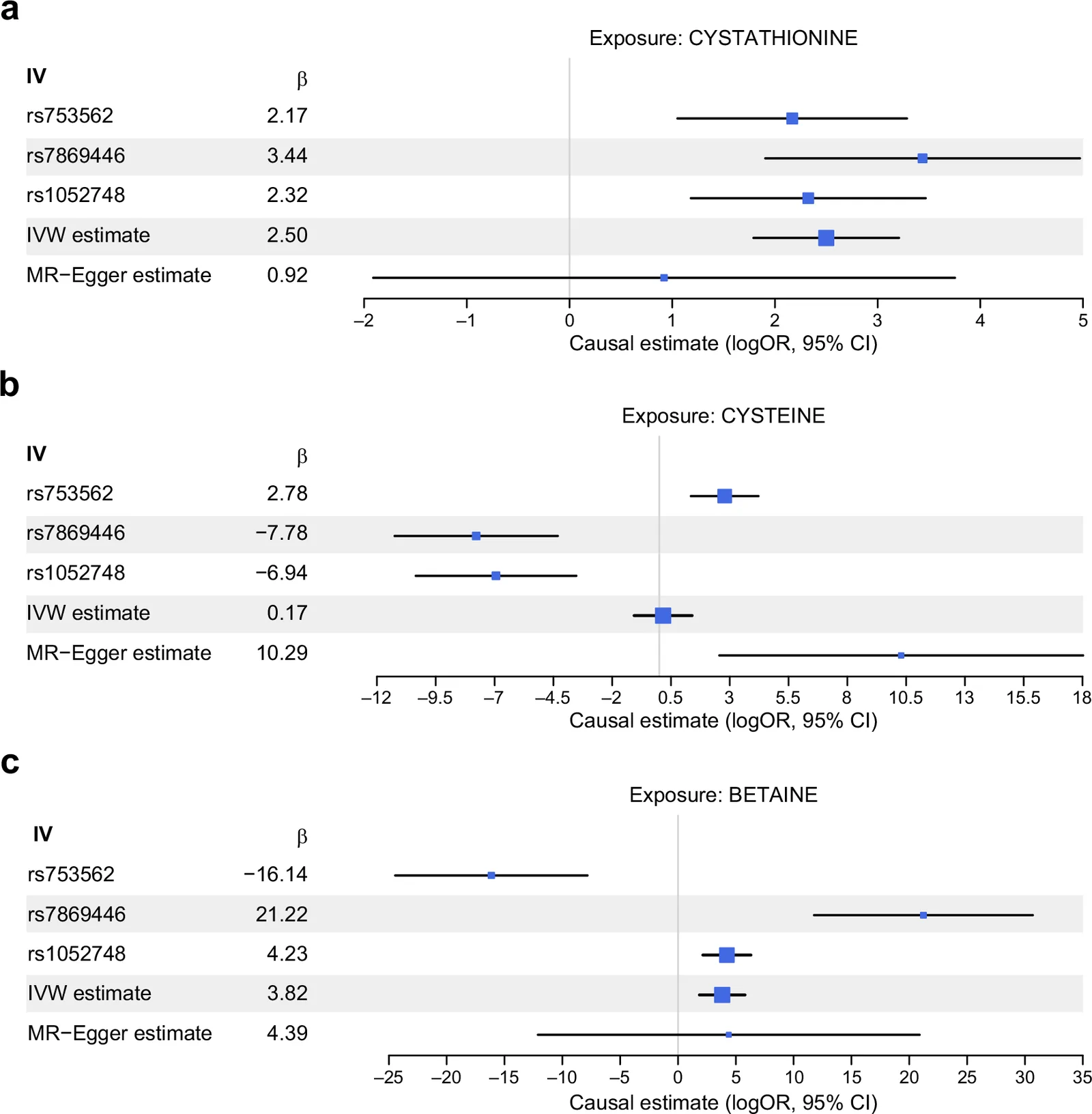
Mendelian Randomization Analysis of OCM SNPs and metabolites in ESAM susceptibility. Causal effects are estimated using a two-sample Mendelian randomization (MR) approach to model combined effects from three independent SNPs. Estimates of SNP–exposure associations are derived from linear regression analyses of serum metabolites adjusted for sex and age; estimates of SNP–disease associations are obtained from GEMMA analyses of the Malawi group in the primary cohort. Both IVW and MR–Egger tests are two-sided and no adjustment for multiple comparisons was applied. The MR–Egger intercept test was used to assess directional pleiotropy. In all panels (**a**–**c**), point estimates represent the log-odds ratios from MR analyses, and error bars indicate 95% confidence intervals centered on these estimates. **a** Forest plot of causal SNP effects on cystathionine in the risk of developing ESAM vs NESAM. The MR-Egger estimate is reported as log-odds ratio *p* = 0.92, *p* = 0.52. IVW estimate for log-odds ratio = 2.50, *p* = 1.65 × 10^–6^, heterogeneity test statistics (Cochran’s Q test *p* = 0.39). **b** Forest plot of causal SNP effects of cysteine. The MR-Egger estimate is reported as log-odds ratio = 10.29, *p* = 0.009. IVW estimate for log-odds ratio = 0.17, *p* = 0.79, heterogeneity test statistics (Cochren’s Q test *p* = 1.66 × 10^–11^). **c** Forest plot of causal SNP effects on betaine. The MR-Egger estimate is reported as log-odds ratio = 4.39, *p* = 0.60. IVW estimate for log-odds ratio = 3.82, *p* = 0.0001, heterogeneity test statistics (Cochren’s Q test *p* = 2.16 × 10^–08^). IVW inverse variance weighted method, CI confidence interval, OR odds ratio.

We first assessed SNP-metabolite and SNP-disease associations separately under regression models and then combined effects using the IVW method, assuming additive genetic effects. Although OCM metabolites are highly correlated, we found significant causal effects on cystathionine (log-odds ratio = 2.50, two-sided *p* = 1.65 × 10^−6^) and on betaine (log-odds ratio = 3.82, two-sided *p* = 0.0001) (Fig. 4). With three SNPs, there was substantial genotypic heterogeneity in the causal effect of betaine and cysteine (two-sided *p* = 2.16 × 10^–8^ for betaine and 1.66 × 10^–11^ for cysteine by Cochran’s Q test for both). We thus reevaluated the model after excluding any SNPs with opposing effects (although such effects could reflect the effect of the major allele), which necessarily limited our analyses to the IVW approach. Using the two SNPs with congruent effects, the causal effect of cysteine was estimated as –7.97, (two-sided *p* = 2.24 × 10^–6^ and Cochran’s Q test *p* = 0.74) and the estimate for betaine was 5.41 (two-sided *p* = 0.0001 and Cochran’s Q test *p* = 0.005). Although we did not find strong evidence for cystathionine or betaine under the MR-Egger approach, we found that estimates from both approaches were similar in direction and magnitude.

### Population ancestry influences the association of OCM loci with ESAM

In the absence of an available secondary cohort in which to replicate our findings, we also sought evidence of internal consistency in the magnitude and direction of effect between the two countries represented in our cohort. At our six candidate loci, minor allele frequencies (MAFs) and directions of effect among associated SNPs were similar between the two countries (Supplementary Data 5). Despite similarities in the direction of effect in the two countries, we noted that the magnitude of the effect was generally stronger in Malawi than Jamaica despite comparable sample sizes and minor allele frequencies (Supplementary Fig. 3). This difference was also evident when we looked at statistically inferred haplotypes (“Methods”) comprised of associated (genotyped) SNPs at our two top loci—*GABBR2* and *PRICKLE2*. We observed evidence of association with ESAM in each population group; however, the haplotypes with the strongest implied effect differed between the two population groups despite similar patterns of LD in the two groups at both loci (Supplementary Fig. 4). Similarly, when we stratified our cumulative association analyses by population group, there was always a higher proportion of association SNPs in Malawi (Fig. 3c).

**Fig. 5:**
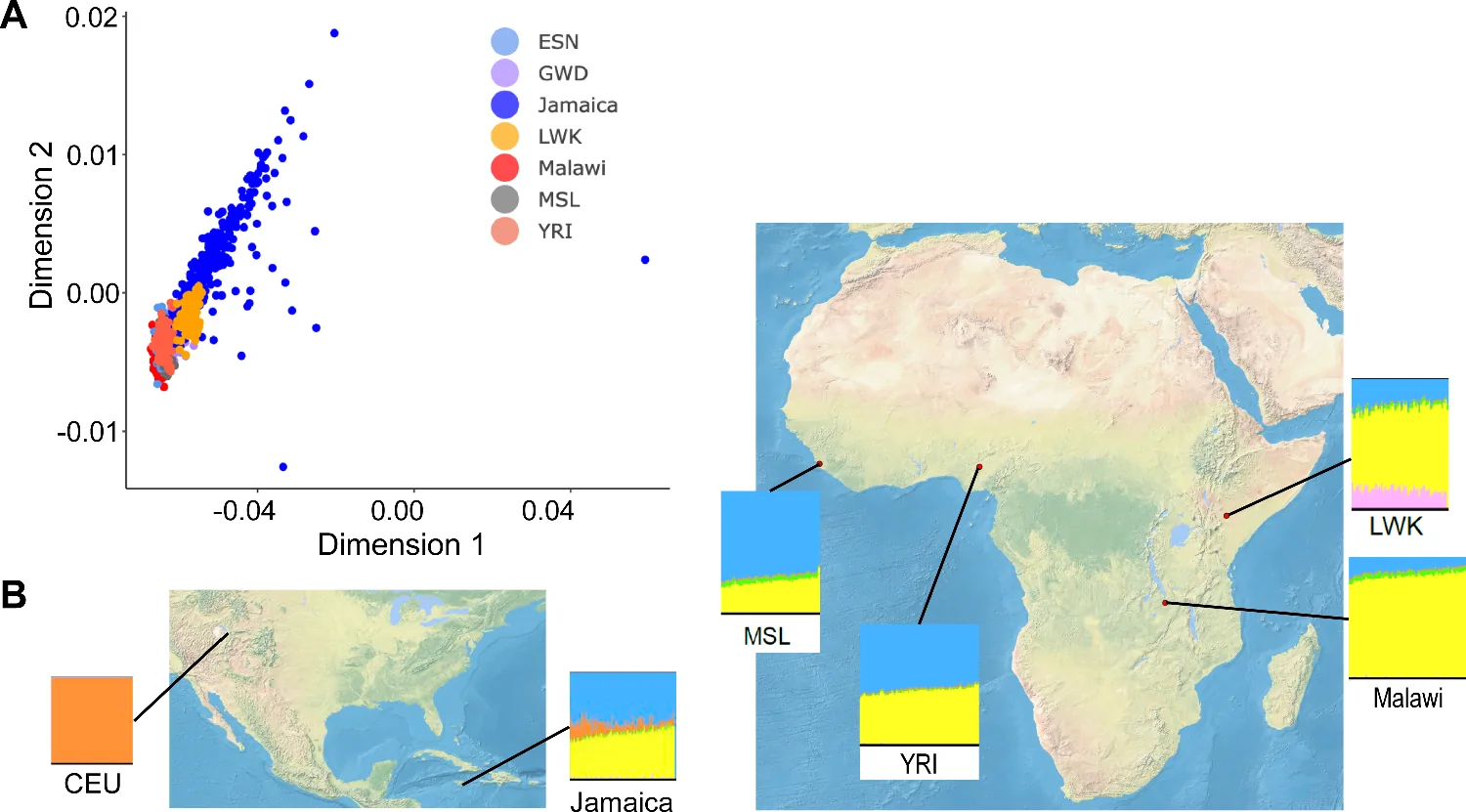
Admixture and ancestry in the SAM cohort. **a** Multidimensional scaling dimensions (C1 vs C2) for Jamaican and Malawian SAM cohort in the context of African (AFR) populations from the 1000 genomes project (ESN Esan in Nigeria, GWD Gambians from Western Division, LWK Luhya in Webuye, Kenya, MSL Mende in Sierra Leone, YRI Yoruba in Ibadan, Nigeria). **b** ADMIXTURE plots (K = 5) for Jamaicans and Malawians in the SAM cohort shown in geographical context of putative ancestral parent populations across Africa and North America (CEU – Utah Residents (CEPH) with Northern and Western European ancestry; LWK Luhya in Webuye, Kenya, MSL Mende in Sierra Leone, YRI Yoruba in Ibadan, Nigeria). Maps were generated using open source QGIS Geographic Information System (see Data Availability), accessed May 2026 with Natural Earth as the basemap.

To explore this further, we first considered our populations in multidimensional scaling (MDS) space. Jamaican and Malawian samples clustered with other African continental populations on MDS components one and two, although samples from Malawi were tightly clustered alongside other Bantu-speaking Niger Congo ethnolinguistic groups (YRI, MSL). Conversely, Jamaican samples were more dispersed (Fig. 5a**;** Supplementary Fig. 5), likely a result of the diverse population ancestry origins of Jamaicans, which include a predominant contribution of African ancestry from the British slave trade of the 14th to 17th centuries, as well as a subsequent influx of indentured peoples from East- and Southeast- Asia, and Europe, in addition to centuries of European (British and Spanish) colonialization.

We then used ADMIXTURE^44^ to infer patterns of genome-wide admixture in our cohort in the context of parental proxies of geographic and continental differentiation derived from the African Genome Variation Project (AGVP)^45^, the Human Health and Heredity in Africa (H3Africa) Project^46^, and 1000 Genomes (1KG) Project^47^ (“Methods”). In this analysis, we proxied European ancestry through the Ceph from Utah (CEU); West African ancestries through the Yoruba from Nigeria (YRI) and Mende from Sierra Leone (MSL); East African ancestries through the Luhya from Webuye, Kenya, and the Amhara from Ethiopia; and southern African ancestries through individuals from Botswana (BOT), Bantu-speakers from Zambia (BSZ), the Zulu, and Khoe and San Hunter-Gatherer groups (KS) (Supplementary Fig. 6). As expected, samples from Jamaica had, on average, a significantly higher proportion of European ( ~ 13%) and West African ancestry ( ~ 68%) than Malawians, in whom East-African ancestries ( ~ 83%) were more common (Welch two-sample t-test, two-sided *p* = 2.2 × 10^–16^; West African comparison) (Fig. 5b).

### Association between OCM loci and ESAM is driven by shared East-African ancestry

Given the ancestral genetic differences between the two study populations, concomitant differences in associated haplotype backgrounds, and reported geographical variation in ESAM prevalence, we considered that the underlying OCM-ESAM association might be best represented by an ancestral haplotype that is shared by the two populations but seen more commonly in Malawi. In this shared ancestry model, the presence of additional ancestry backgrounds (admixture) might obscure the underlying association signal, especially if using genome-averaged ancestry to account for population stratification. To evaluate this, we used RFMix^48^ to derive ‘local’ (locus-level) patterns of ancestry, using the CEU, MSL, and LWK populations to proxy European, West African-, and East African- Bantu-speaking parental ancestries, respectively, in our cohort (“Methods”). We then repeated our OCM-wide association tests, but this time adjusting each SNP for its ancestral haplotype background using TRACTOR. This approach allowed us to account for the potential effects of differing ancestral haplotype backgrounds at putatively associated loci in a way that was agnostic to geography but still ancestrally sensitive.

**Fig. 6:**
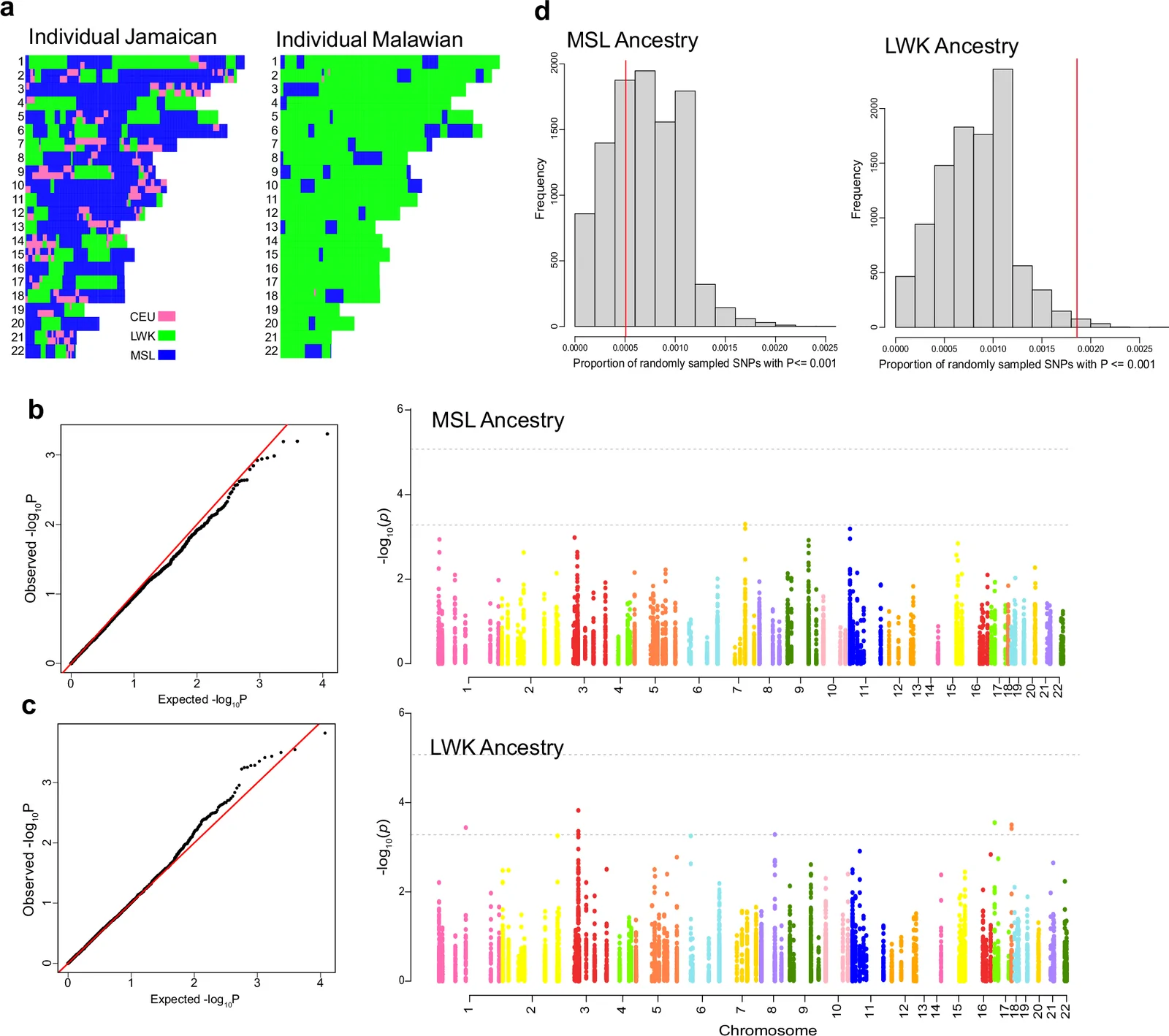
Local ancestry association. **a** illustrative local ancestry plots for Jamaican and Malawian individuals. Three letter codes refer to populations from the 1000 genomes project: CEU Utah Residents (CEPH) with Northern and Western European ancestry; MSL Mende in Sierra Leone, LWK Luhya in Webuye, Kenya. **b** QQ plot and Manhattan plot for TRACTOR-based association conditioning on West African (MSL) ancestry. **c** QQ plot and Manhattan plot for TRACTOR-based association, conditioned on East African (LWK) ancestry. For both **b** and **c**, association testing was performed using the TRACTOR framework with a logistic regression model. All tests were two-sided. To account for multiple comparisons, Bonferroni corrected genome-wide significance thresholds are indicated. **d** Cumulative association (see methods) by ancestry group showing the empirical distributions of the proportion of 5916 SNP associations in TRACTOR with *p* < 0.001 after resampling 10,000 times. The red line shows the observed proportions for 5916 SNPs at OCM loci. Statistical significance was assessed using an empirical permutation test (one-sided), with *p* values calculated as the proportion of permutations exceeding the observed statistic. No multiple-comparison adjustment was applied.

Consistent with genome-wide admixture estimates, Jamaican individuals harbored more blocks of European- and west-African- derived ancestry than Malawi (Fig. 6a). Across the combined cohort, however, the overall ancestry proportions in ESAM were not significantly different than seen in the reference NESAM population (t-test, lowest two-sided *p* = 0.24; Supplementary Fig. 7), suggesting that differences in effect size and association in our original association were not solely the consequence of mismatched ancestry between ESAM and NESAM. Using TRACTOR to perform logistic regression with 3-way admixture, we found that when conditioned on shared East African ancestry (proxied by LWK), seven loci surpassed the loci-level threshold for association, including candidates *PRICKLE2*, and *PLD2*; however, only one OCM locus, and none of our candidates, met the same significance criterion when adjusting for West African ancestry (Fig. 6b, c). We also observed that stratifying our random sampling cumulative association by ancestry background demonstrated a higher cumulative association between variants of presumed East African ancestry compared to West African ancestry at a *p* value threshold of 0.001 (z = 2.81 and z = 0.54, respectively) (Fig. 6d). Collectively, these findings were consistent with our OCM-ESAM association being driven by variants carried on a shared haplotype background more common among individuals of East African ancestry.

### Signatures of selection at OCM loci among African populations

Periods of famine have been associated with substantial childhood mortality, with survival into adolescence following childhood starvation potentially having a major effect on reproductive fitness. In the absence of modern-day nutritional support, ESAM is associated with higher mortality than NESAM, creating the potential for variants and loci that influence the risk of developing ESAM or NESAM to be subject to relatively strong selection. To explore this further, we generated haplotype-similarity-based scores (iHS) of recent selection at our OCM loci (±50 kb) using whole genome sequence data from a subset of 151 individuals from Malawi (“Methods”). We contextualized our results by considering iHS scores for the same OCM loci generated from genome sequencing done through the H3Africa consortium^46^, which includes samples from seven African countries: Benin, Botswana, Cameroon, Mali, Nigeria, Zambia, and Gur speakers from West Africa (Burkina Faso and Ghana).

**Fig. 7:**
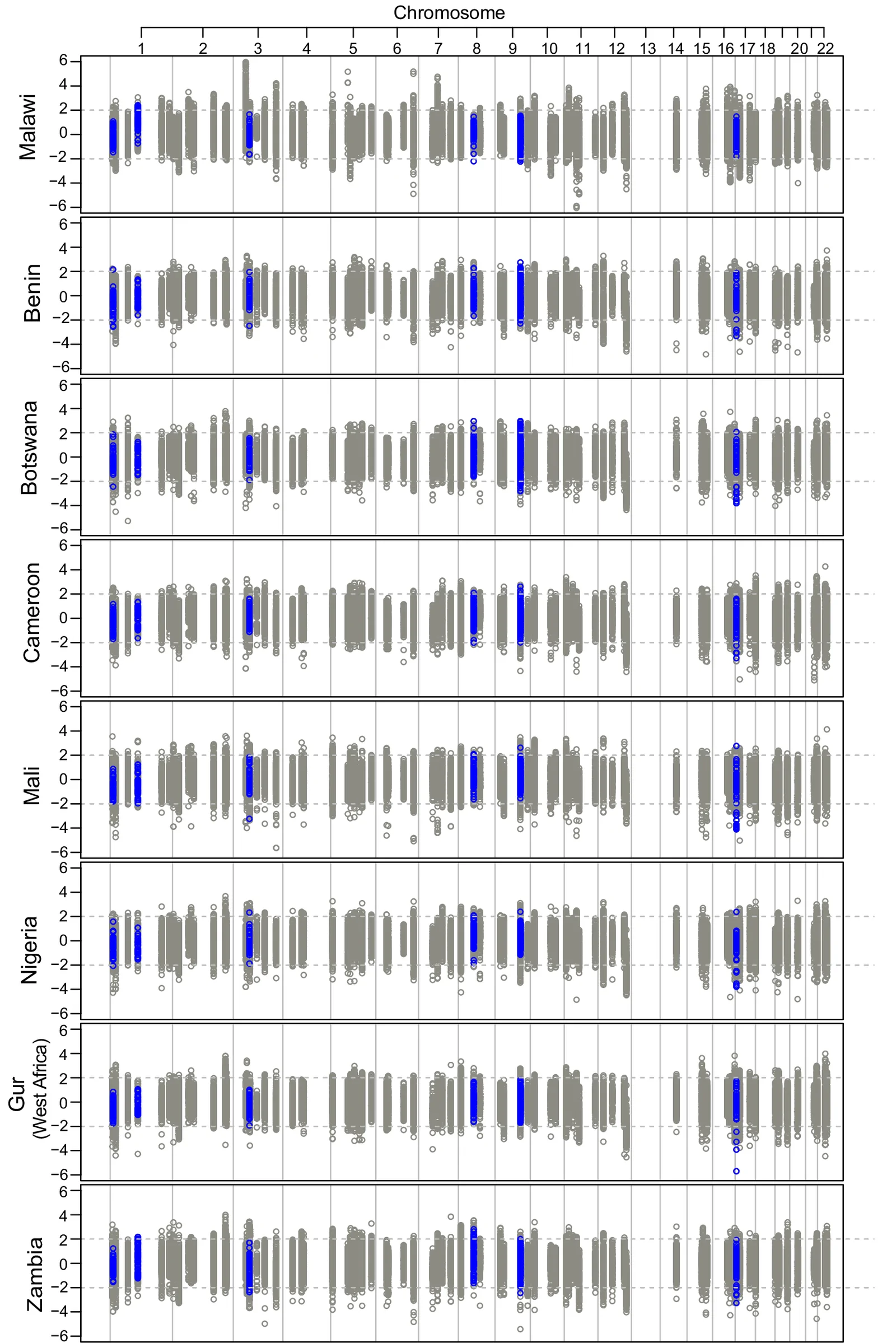
Standardized iHS scores for OCM loci across Africa. Gray open circles identify iHS scores within 50 kb of OCM genetic loci. Blue circles indicate iHS scores in regions within 10 kb of ESAM-associated OCM genetic loci. The dashed lines represent thresholds of +2 and −2 iHS scores, which are suggestive of strong selection. There were no included OCM loci on chromosome 13.

Using an ad hoc, but conservative, locus-selection threshold of more than 10% of SNPs having normalized iHS scores >2 or <–2, we found that in Malawi, 15 of our 95 OCM loci (15.8%) had some evidence of selection, with *AMT* (0.429), *SDS*/*SDSL* (0.301), *MDH2* (0.208) and *SHMT1* (0.174) having the most SNPs surpassing our threshold (Supplementary Data 6). Of these loci, however, none overlapped our top ESAM-associated candidate loci, most of which had proportions <3%. Across the African countries in the H3Africa dataset, however, 2440 SNPs within 50 kb of OCM genes had evidence of selection in at least two countries (Supplementary Data 6**;** Supplementary Fig. 8), with 55 SNPs having strong evidence across all surveyed groups (Fig. 7). In this broader dataset, rs1052748 (ESAM-associated *PLD2* coding variant) had the strongest selection signal among ESAM-associated OCM SNPs (Fig. 7**;** Supplementary Data 7), as did nearby *PLD2* SNPs, particularly in Zambia (proportion= 9.6%), Cameroon (9.3%), and among Gur speakers from West Africa (13.3%).

Lastly, we considered that haplotype similarity scores at selected loci might be amplified among ESAM samples, mimicking the cumulative allelic effects seen in our association models (i.e. enriching for multiple copies of a risk/protective allele). Across all OCM loci, the mean iHS score was significantly higher in ESAM (*n* = 90) than NESAM individuals (*n* = 61) (normalized iHS- two sample *t* test two-sided *p* = 4 × 10^–7^; raw iHS - two-sided *p* = 5 × 10^–14^; Supplementary Fig. 9a). Among variant sites within 10 kb of SNPs surpassing our locus-wide cut-off (*n* = 839 ESAM; *n* = 753 NESAM), mean iHS scores were also significantly different (two sample *t* test two-sided *p* = 0.01; Supplementary Fig. 9b) between the two groups.

## Discussion

We report results of a case-control genetic association study of ESAM (affected/cases) versus NESAM (reference/controls) at loci associated with OCM. To our knowledge, this is the largest genetic study of ESAM to date. Our hypothesis-driven targeted pathway approach allowed us to maximize our modest sample size and, in the absence of an available replication cohort, we find strong internal consistency between the two country cohorts assessed. SAM is an acute, life-threatening disorder, for which, traditionally, recruitment, consent, and sampling for large-scale research are secondary considerations; however, the ability to retrospectively recruit individuals several months to years after surviving SAM holds potential for future, larger, germline genetic studies of ESAM. Here, we report evidence of an association between cumulative and locus-specific genetic variation at OCM-loci and the risk of developing ESAM. This association appears to be driven by variants carried on an East African genetic background and is bolstered by signatures of recent selection at ESAM-OCM-associated loci and by evidence that associated variants partially mediate the association between OCM metabolites and ESAM, although their effects are unlikely to be sufficient to cause ESAM.

Intronic SNPs in *GABBR2* and *PRICKLE2* showed the strongest locus-specific association. Both loci include multiple sites of reported open chromatin, suggestive of regulatory effects; however, it is unknown whether these effects would be limited to the most proximal genes versus having more distal effects on other *cis-* or *trans-* genes. The latter observation is particularly relevant at *GABBR2* where the top SNP (rs7038285) had a much higher minor allele frequency among African populations. The relative lack of transcriptional or gene-regulatory contexts from Africa makes it difficult to fully annotate the regulatory potential of candidate variants identified in studies such as ours. We did, however, observe locus-wide positive association between a GTEx-reported eQTL missense variant in *PLD2* (rs1052748) and ESAM. The minor allele of rs1052748 is associated with reduced *PLD2* transcription. The functional consequence of variable transcription on phospholipase D2 phosphatidyl activity and production of choline from phosphatidylcholine is uncertain; however, reduced choline availability would be consistent with lower choline concentrations noted in ESAM^30^.

Single locus metabolic effects do not occur in isolation - small changes in metabolic flux at multiple points in OCM could result in larger effects on OCM consistent with those reported in ESAM. This was consistent with both our cumulative association model and our Mendelian Randomization models. For instance, most of the loci reaching locus-wide association were associated with homocysteine metabolism, and MR analyses suggested mediating effects of SNPs at *GABBR2, PRICKLE2*, and *PLD2* on metabolites that are either directly derived from homocysteine (Cysteine and Cystathionine) or are involved in its re-conversion to methionine (Betaine). These observations strengthen previous studies implicating aberrant OCM, and particularly deficient re-methylation of homocysteine to methionine, in the pathogenesis of ESAM. These data thus provide support for proposed therapeutic interventions to supplement OCM turnover in SAM using cofactors such as choline (clinicaltrials.gov ID NCT06154174). Despite this, our preliminary estimates (calculated using Genome-wide Complex Trait Analysis (GCTA)^49^) suggest that genetic variation has a moderate effect on the variance in SAM outcomes (estimated heritability= 0.22; standard error (se)= 0.37), variation at OCM loci accounts for a much smaller proportion (estimated heritability= 0.06; se= 0.06). The sample size employed means that these heritability estimates have a relatively large standard error as do the effect sizes inferred from our MR analysis. Optimistically, however, there may be several unidentified loci that causally influence ESAM risk. As a proof-of-principle, we also reviewed the genome-wide association statistics for SNP-association with ESAM; consistent with our use of sample size appropriate to targeting OCM loci, we did not identify any large-effect genome-wide loci with SNPs exceeding genome-wide significance (*p* < 5 × 10^–8^**;** Supplementary Fig. 10; Supplementary Data 8).

Our previous epigenomics study noted overlap between ESAM differentially methylated genes and genes associated with general anthropometric parameters. We thus sought to evaluate our OCM SNP regions for co-association between ESAM and body anthropometry; however, only ten of the 364 genome-wide significant variants associated with waist-to-hip ratio^50^, and none of the 19 genome-wide significant variants associated with body size (obese vs thin individuals)^51^, overlapped our OCM regions, and none of the overlapping SNPs showed evidence of association with ESAM. It may be that OCM loci do not have the same direct effect on anthropometry that they do on ESAM, although we cannot discount that an appropriately powered GWAS of ESAM could still identify co-localizing SNPs that impact both phenotypes. Genome-wide studies of ESAM (and related phenotypes) would be well served by the development of larger cohorts, which we have spearheaded through the establishment of the Kwashiorkor Study Network (KwashNet—see Data Availability). KwashNet includes multiple study sites in areas where the prevalence of ESAM relative to NESAM remains high. We anticipate that this framework will allow for well-powered genome-wide approaches that can identify other ESAM-associated loci and/or pathways for therapeutic and nutritional targeting.

We were able to uniquely leverage African intra-continental admixture patterns to aid in our mapping. The deep ancestral tree within Africa harbors a complex and diverse genetic landscape that can be as disparate as inter-continental variation. The higher rates of ESAM in east-central and southern Africa, therefore, presented an opportunity to employ admixture mapping approaches that have been long proposed for diseases with different population prevalences. We observed evidence of ESAM association at OCM loci when conditioned on East African, but not West African, haplotype backgrounds shared between Malawi and Jamaica. We hypothesized that reducing the ‘noise’ of West African haplotypes in the analysis, would have augmented the effect of shared East African ancestry beyond the effect seen in our primary association. The weaker signal observed likely represents limitations in the representation of African haplotypes in current imputation panels and public databases^52,53^, making imputation and selection of parental ancestral proxies necessarily deficient (e.g., the LWK are an imperfect proxy for the ancestral East African ancestry of Malawi). None the less, our results provide a conceptual model for leveraging admixture within genetically heterogeneous groups to genetically map traits with prevalence differences between groups, provided appropriate parental proxies can be identified and there is sufficient genetic distance between the groups in question.

There are several examples of how migration across, into, and out of Africa, has interfaced with varying infectious and non-infectious exposures to shape the African genome through adaptation^54^. More recent studies have implicated multiple novel loci, often with varying effect sizes across the continent^46^. The history of famine and food crises in Africa varies by country and are complex; periods of famine have been documented as far back as the 1600s^55^, with speculation that these were more severe in the 19^th^ century, at least in east and southern African countries such as Zimbabwe and Tanzania^56,57^. At the same time, children developing ESAM have been noted to have higher birth weights^58^ and divergent cardiometabolic outcomes relative to NESAM in adulthood^59,60^. The latter observations have typically been attributed to materno-fetal exposures *in utero* (Barker Hypothesis), although our genetic results suggest there may be previously unrecognized gene-x-environment effects. Differential survival between ESAM and NESAM could be pleiotropic—offset by advantageous effects in other contexts - and reflected in signatures of recent selection at ESAM-associated OCM loci. Generally, several OCM loci had strong evidence of selection, particularly the serine dehydratase (SDS/SDSL) complex on chromosome 12, which had a strong signal in all the populations evaluated. Among our ESAM-associated OCM loci, however, only *PLD2* showed evidence of selection. Like our genetic association, we observed that OCM selection scores among ESAM individuals were, on average, significantly higher than observed among participants with NESAM, suggesting that we may be similarly underpowered to observe subtle selection signatures at individual loci that contribute to a pathway-wide metabolic effect. Going forward, integration of selection statistics at scale, particularly in the context of African genomic association studies, could provide valuable biological insights, especially for novel loci and understudied disorders.

Our results provide a unique view of the SAM-NESAM dichotomy; however, there are limitations to our study. Aside from the sample size limitations already noted, we were limited in our coverage of genomic variation. The H3Africa SNP array offers a robust coverage of LD among genetically African ancestries; however, it is far from fully comprehensive. Although genome sequencing efforts in Africa (and among populations of predominant African ancestry) are increasing; the number of reference genomes, particularly for disparate African groups, is still much smaller than that available for European ancestry groups. As a result, accurate and confident imputation, and parental ancestry mapping were particularly challenging in our study, and may have led to false negative or overly conservative results. This highlights the need for larger and more extensive genetic databases of sequence variation from a broad cross-section of African populations, particularly from foundational groups with unique ancestries. Subsequent integration and curation of the derived data into reference panels and widely accessible databases of African genetic variation will facilitate more robust studies.

Genome sequencing and genetic mapping efforts in Africa and among populations of predominantly African ancestry are gaining traction, although the number of reference genomes, particularly for non-West African groups, still vastly under-represents the variation across the continent. Our study provides a starting exemplar for applying a hypothesis-driven, population genetics-informed model to study a disease with high prevalence in specific African population groups. Continued expansion of sequencing efforts in African populations, alongside refinement and deployment of association models that can integrate intra-continental admixture and population genetic statistics, has the potential to maximize the future of human genetics studies on the continent and globally.

## Methods

### Study participants

Details of participant samples used in the study have been previously published^33,37^. All recruitment was conducted in compliance with local Ethics Committees and Institutional Review Boards (IRBs) at UHWI/University of the West Indies Faculty of Medical Sciences, the National Health Science Review Committee of the Ministry of Health, Government of Malawi, and through Baylor College of Medicine (BCM) as outlined below. All samples were collected after informed consent was obtained for genetic studies of SAM and participants had received a diagnosis of either ESAM or NESAM.

Samples from Jamaica included DNA from blood and buccal samples from children ( < 18 years) diagnosed with SAM and were obtained for purposes of investigating the genetics of ESAM. DNA was extracted using a phenol/chloroform protocol as described previously^37^. SAM subtype was determined using the Wellcome Classification^61^, where NESAM (marasmus) is defined as <60% weight-for-age without edema, and ESAM includes marasmic-kwashiorkor- defined as <60% weight-for-age with edema, and kwashiorkor - defined as 60–80% weight-for-age with edema. Pediatric participants were recruited prospectively from the Tropical Metabolism Research Unit of the Caribbean Institute for Health Research at the University Hospital of the West Indies (UHWI), the major tertiary nutritional center on the island. Recruitment was part of an ongoing study of the genetic etiology of SAM. A cohort of adult participants ( > 18 years) who formerly had SAM as children were recruited from the same site for the same purposes. The study was approved by the Ethics Committees of the UHWI/University of the West Indies Faculty of Medical Sciences.

In Malawi, samples from children with SAM were recruited from 18 rural sites across five districts^33^. Samples were collected between 2013 and 2016, originally for genetic studies of kwashiorkor, which included the current study. The sub-type of SAM was defined using mid-upper arm circumference (MUAC) and the presence or absence of edema. Buccal samples were collected using the Oragene Discover (OGR-250) DNA collection kit (DNA Genotek Inc., Ottawa, Ontario, Canada). The protocol was modified to collect buccal epithelial cells by swabbing the inside of each cheek ten times. DNA was extracted following the manufacturer’s instructions. After collection, to maintain clinical uniformity in SAM designation between Jamaica and Malawi, samples from Malawi were re-classified using the Wellcome Classification as having either ESAM or NESAM, which was highly concordant with the MUAC based designation, with the few participants reclassified as moderate acute malnutrition being removed. The study was approved by the National Health Science Review Committee of the Ministry of Health, Government of Malawi.

Written informed consent was obtained from the parents or adult guardians of children included in this study. Permission to use de-identified participant samples for genetic studies at Baylor College of Medicine (BCM) was approved by the Institutional Review Board (IRB) of BCM (IRB# H-27090) and confirmed by the National Institutes of Health IRB subsequently.

### Curation of OCM loci

GeneWeaver^62^, AmiGO 2^63,64^, GWAS Catalog^65,66^, KEGG^67^, NCBI BioSystems^68^, Pathway Commons^69^, and PubMed were queried using the search terms “one-carbon metabolism”, “cysteine”, “methionine”, and “one-carbon pool by folate” to identify OCM loci to be used in this study. At the genome level, a 50 kilobase (kb) buffer region was included on either side of each identified gene to capture potential *cis*-acting elements. This search resulted in a list of 103 genes involved in OCM. Overlapping regions were consolidated into single regions, resulting in 95 loci (Supplementary Data 2).

### Genotyping and quality control

Peripheral blood and buccal DNA samples from Jamaican and Malawian individuals were genotyped for this study. Downstream quality control and analyses are shown in Supplementary Fig. 1. Genotyping was performed on the Infinium H3Africa Consortium Array v1.1 (**web resources**), which provides LD coverage of ~0.89 in the African superpopulation of 1000 Genomes. Raw idat files were processed in GenomeStudio (Illumina, San Diego, California, USA), including within-sample clustering, to create binary input files for PLINK. Genotype fluorescence projections in GenomeStudio were visually inspected for those SNPs that surpassed test-wide significance in order to confirm genotype calls. Downstream SNP and sample quality control (QC) was performed in PLINK v1.07^70^ and included limiting to biallelic autosomal SNPs and excluding SNPs with a missingness >0.05; removal of SNPs outside the Hardy-Weinberg test distribution; and excluding samples with missingness >10%. Linkage disequilibrium (LD) pruning was conducted using a window size of 50 bases, a sliding window of ten bases, and r^2^ threshold of 0.1. Pairs of samples with a proportion-of- identity-by-descent (IBD) (PI_HAT) score >0.1 were flagged, and the individual within the pair with the higher missingness rate was removed. Individuals with conflicting chromosomal and reported sex were reevaluated for potential sample swaps and flagged for removal if not resolved. Post QC, 1.8 million genome-wide genotyped SNPs were available for downstream analysis across the 711 remaining samples.

### Imputation

Given the relative lack of representation of African genomes in existing imputation panels, we chose to conduct a two-stage imputation and data merge process (Fig. 1; Supplementary Fig. 1). Data from Jamaican individuals (*n* = 340) were imputed using the Consortium on Asthma among African-ancestry Populations in the Americas (CAAPA) reference panel^71^, which was generated using data from individuals with predominantly West-African ancestry, including Jamaicans. We used minimac3 v1.0.5 (University of Michigan, Ann Arbor, Michigan, USA)^72^ and Eagle v2.3 (Broad Institute, Cambridge, Massachusetts, USA)^73^ on the Michigan Imputation Server (University of Michigan, Ann Arbor, Michigan, USA)^72^ to perform phasing and imputation on our Jamaican samples, selecting the African reference panel (“AFR”) as the reference population.

Data from Malawian participants (*n* = 371) was imputed using the H3Africa reference panel (H3ABioNet)^74^ (**web resources**), which is designed to represent a wide array of African ancestries, including east- and central-African groups that neighbor Malawi. Phasing was performed using Eagle 2.4^73^ (Broad Institute, Cambridge, MA, USA). Minimac4 Version 1.0.0 (University of Michigan, Ann Arbor, MI, USA) was used for imputation on the H3ABioNet Nextflow chip imputation pipeline^75^, which implements a similar workflow to the Michigan Imputation Service pipeline, and was deployed on the University of Cape Town High-Performance Computing Facility (web resources).

After imputation, SNPs with r^2^ < 0.3 were excluded. The imputed data from both population groups was merged using an in-house command line script (**web resources**), prior to removing non-autosomal SNPs and SNPs with minor allele frequency (MAF) < 0.05. The final imputed dataset included 8,415,377 SNPs, of which 45,411 SNPs were at OCM loci (±50kb of genes).

### Multidimensional scaling

Genome-wide SNPs were used to perform multidimensional scaling (MDS) in PLINK v1.07, and these were used for two different purposes (see Supplementary Fig. 1): 1. on the QC-ed genotype dataset used for association analyses; 2. on the genotype-only dataset alongside datasets from 1000 Genomes^47^ to put the primary populations in the context of African datasets (See Population ancestry and admixture mapping below). 1000 Genomes datasets consisted of Esan individuals in Nigeria (ESN, *n* = 99), individuals from Gambia in Western Division, the Gambia (GWD, *n* = 113), Luhya individuals in Webuye, Kenya (LWK, *n* = 99), Mende individuals in Sierra Leone (MSL, *n* = 85), and Yoruba individuals in Ibadan, Nigeria (YRI, *n* = 108). The genotyped-only dataset was merged with the above 1000 Genomes populations, and LD-pruned with a window size of 50 bases, a window of 10 bases, and r^2^ < 0.1. MDS was performed PLINK and visualized using an in-house R^76^ script (Supplementary Fig. 5).

### Genetic association analyses

Association statistics were primarily derived using Genome-Wide Efficient Mixed Model Association (GEMMA)^77^, which better accounts for genetic ancestry variation. Mixed model association analysis in GEMMA was performed using the -lm 4 flag. Standardized relatedness of the combined genotyped and imputed dataset was also calculated using the flag -gk 2. We used the genetic data to estimate the standardized kinship matrix before applying the generalized linear mixed effect model to assess genetic association between SNPs and the risk of developing ESAM, assuming random effects on individuals and fixed effects due to sex and the first component of MDS (as suggested by scree plots of the proportion of variance explained; Supplementary Fig. 11). In the regional regression analyses, the MDS component was estimated from the dataset of the specified cohort. QQ plots were generated using the R package qqman^78^. Manhattan plots were generated using an in-house script (**web resources**).

### Cumulative association

We employed a permutation test procedure to assess the cumulative association between OCM loci and SAM status; this is a non-parametric method of assessing the statistical significance of observed data without making assumptions about the underlying probability distribution of the data. We hypothesized that OCM loci are enriched for SNPs with *p* values below a certain significance threshold, i.e., the proportion of significant loci within OCM loci is more than expected by chance. We choose *p* = 0.001 as a representative significance threshold for the number of loci being tested (similar to a Bonferroni correction for multiple testing of 0.1/100). To test the robustness of our findings, we included a ceiling threshold of *p* = 0.01, and two equidistance points as sensitivity points.

The test procedure involved taking the genome-wide association statistics for all genotyped SNPs (*n* = 1,843,153) and calculating the proportion of SNPs with a *p* value meeting a pre-specified threshold. We then randomly sampled a subset of SNPs equivalent to the number of genotyped SNPs across OCM loci (*n* = 9479) and determined the proportion of those SNPs with a *p* value meeting the predefined threshold. That step was repeated 10,000 times to derive an empirical distribution of the proportion of significant SNPs. We then calculated a z-score for the observed proportion based on the empirical distribution. To account for the randomness of genome-wide SNPs relative to a restricted metabolic pathway, we performed the same test using a similar number of SNPs sampled from loci involved in ketone metabolism.

### Haplotype association and calculation of linkage disequilibrium

Genotyped SNPs in *GABBR2* (*n* = 5) and *PRICKLE2* (*n* = 6) reaching region-level significance (Fig. 2) were arranged in 5’ to 3’ order and phased in PLINK v1.07 to create basic region-level haplotypes. PLINK was also used to determine association using the --hap-assoc flag. PLINK files were also parsed to Haploview^79^ in order to generate plots of linkage disequilibrium.

### Population ancestry and admixture mapping

To investigate the ancestral African genetic ancestries in the Jamaica and Malawi datasets, genome-wide genotype data was merged with selected population data from publicly available datasets using PLINK v1.9 (Fig. 1; Supplementary Fig. 1). Available datasets included the African Genome Variation Project (AGVP)^45^, H3Africa Consortium^46^, Schlebusch et al.^80^, and 1000 Genomes Project (Phase III). Only SNPs common to all datasets were retained. As genetic reference data is not available for every African ethnolinguistic group, we sought to ensure that we could assess contributions from Niger-Congo speaking groups, which includes Bantu speakers—the largest ethnolinguistic grouping across the continent, as well as non-Niger-Congo groups, such Rain Forest Forager, Hunter-Gatherer (e.g. Khoe and San), and Nilo-Saharan groups, which are more populous in east- and central- Africa. Populations representing Niger-Congo African ancestry included Bantu speakers from Zambia (BSZ, *n* = 39), Jola (*n* = 79), and YRI (*n* = 100). Populations representing non-Niger-Congo African ancestry included Amhara (*n* = 42), Botswana (BOT, *n* = 48), LWK (*n* = 74), Khoe and San Hunter Gatherers (*n* = 18), Zulu (*n* = 95), and MSL (*n* = 85). Samples from CEU (*n* = 95) were included to represent European admixture. The merged dataset was further filtered to remove SNPs with excessive missingness ( > 0.1) and then LD-pruned (see MDS above). The final merged genotype dataset included ~50,000 autosomal SNPs. The resulting merged dataset was used to perform MDS for a subset of groups using the same parameters and procedures as above.

Using the unsupervised clustering algorithm implemented by ADMIXTURE v1.3^44^, global ancestry proportions were inferred for all populations. Since the algorithm is known to be impacted by the number of individuals that represent a population, 100 random individuals each from Jamaica and Malawi were retained to approximate the sample sizes of the reference populations. ADMIXTURE was performed for K = 2 to K = 6, and the K with the lowest cross-validation error estimates was noted (K = 5).

### Local ancestry inference

Supplementary Fig. 1b illustrates the data processes for local ancestry. Data subset from 1000 Genomes consisting of CEU, MSL, and LWK individuals was used as references for European, West African, and East African ancestry, respectively. The SAM cohort dataset and the 1000 Genomes dataset each underwent independent QC filtering using MAF > 0.05; SNP missingness threshold of 0.05; and removal of duplicate SNPs. The remaining SNPs were merged using an in-house script. The merged dataset was subject to additional QC filtering to remove A > T and G > C SNPs. Datasets were then phased by chromosome using ShapeIt2^81^, using the 1000 Genomes Phase 3 genetic map with default parameters.

Local ancestry inference scores were calculated for the genotyped-only dataset using RFMix version 2^48^. Local ancestry for the reference 1000 Genomes dataset was run separately from the SAM cohort dataset to maximize the available data generated on differing platforms, and 1000 Genomes-specific indices were used as reference ancestry groups. The 1000 Genomes Phase 3 genetic map was used for RFMix, which was otherwise run with default parameters.

### Ancestry-adjusted association analyses

In ancestry-adjusted association tests, we employed TRACTOR v1.1.0^82^. SNP association with ESAM disease risk was performed whilst accounting for the ancestral origin of the SNP by first assigning ancestry labels to the two haplotypes of individuals, referred as tracts. Tracts were extracted from the genotyped-only dataset using ExtractTracts.py from TRACTOR, with integers from one to three referring to the three proxy ancestries—CEU, LWK, and MSL—from 1000 Genomes. RunTractor.py was then run to perform logistic regression using NESAM vs ESAM status as the outcome for each individual, with tracts, ancestry-specific allele counts, and sex as regressors. OCM loci were then subset from the regression results and visualized^78^.

### Cell-free DNA genotyping of serum metabolomics samples

Cell-free DNA (cfDNA) was obtained from serum samples of 415 individuals from Malawi. These individuals were independently recruited as part of our published metabolomics study of one-carbon metabolites in SAM^30^. Samples were initially stored at –80 °C and subsequently slowly brought up to room temperature to minimize degradation. CfDNA was extracted using the Qiagen QIAamp MinElute Virus Spin Kit (Cat #57704), which is optimized for cfDNA isolation, and yielded a mean concentration of 2.7 ng/µL of genomic DNA per sample, with concentrations ranging from 0.1 ng/µL to 52.0 ng/µL.

We successfully amplified four ESAM-associated SNPs from cfDNA samples (Supplementary Data 4)—two in *PRICKLE2* (rs753562 and rs17664202), one in *GABBR2* (rs7869446) and one in *PLD2* (rs1052748). Primers for these SNPs were designed using an in silico primer design tool (https://www.ncbi.nlm.nih.gov/tools/primer-blast/) using the SNP ID (rsID) in GRCh38/hg38 (**Supplementary Information**). Primer specificity was confirmed using BLAT. Primers were synthesized by IDT (Coralville, IA, USA) and prepared as 100 µM stock solutions in TE buffer. PCR amplification was performed using 4 ng of DNA under a single thermocycling and amplification protocol, with products verified via gel electrophoresis (GelDoc, BioRad laboratories, Hercules, CA, USA). Following PCR, products were purified using Exo-Sap IT (Thermo Fisher Scientific, Waltham MA, USA) to remove excess primers and dNTPs. Dideoxy- (Sanger) sequencing was conducted using M13-tagged primers and the Big Dye v3.1 sequencing kit (Thermo Fisher Scientific, Waltham MA, USA). Sequencing reactions were cleaned using the Qiagen DyeEx kit (Qiagen, Germantown, MD, USA) to remove dye terminators, followed by genotype calling on the SeqStudio (Genetic Analyzer #A33770, Fisher Scientific, Waltham MA, USA). Sequencher (software version 4.10.1) was used to visualize and confirm genotype calls.

### Mendelian randomization (MR) using serum metabolites

Following the same classification criterion used in our primary cohort, we selected 252 samples with diagnoses of ESAM (*n* = 135) and NESAM (*n* = 117) for whom we had cfDNA genotypes *and* metabolomic data for 15 OCM metabolites—choline, betaine, dimethylglycine (DMG), glycine, sarcosine, 5-methyltetrahydrofolate (MTHF), serine, methionine, S-adenosylmethionine (SAMe), S-adenosylhomocysteine (SAH), homocysteine, cysteine, cystathionine, pyridoxal phosphate (PLP) and asymmetric dimethylarginine (ADMA). The remaining samples were either community controls or had a diagnosis of moderate acute malnutrition.

Mendelian randomization (MR) was used to assess the causal effect of individual metabolites using the genotypes as instrument variables. For this analysis, we performed two-sample MR based on multiple SNPs by combining results from the SNP-metabolite and the SNP-outcome associations to show potential in vivo effects. We applied one instance of the inverse-variance approach (IVW) from MendelianRandomization R package. Under this approach, the causal effect is obtained from a weighted linear regression of the associations with the outcome, on the associations with the metabolite, while fixing the intercept to zero and weights being the inverse-variances of the associations with the outcome^83^. We also exploited the MR-Egger method to detect invalid instrument variables and bias^84^. The serum metabolites were highly correlated (R^2^). Our cumulative association data suggested that there might be synergistic effects among SNPs; therefore, we assumed an additive genetic model for test alleles, and then tested for the causal effect on ESAM risk of each metabolite, after log_10_ transformation (to better approximate normality). We selected three genotyped SNPs as instrument variables, one SNP from each locus, with SNP rs753562 in gene *PRICKLE2* being omitted because the serum genotype data was slightly out of Hardy-Weinberg equilibrium and the SNP is physically close to the other *PRICKLE2* SNP. The estimates of SNP-outcome association are drawn from the GEMMA analysis of the Malawi group in the primary cohort with fixed effects being sex and the first component of MDS. The estimates of SNP-metabolite association are drawn from a linear regression of log_10_(metabolite) with sex and age as covariates.

### Whole genome sequencing and signatures of selection

A subset of 151 DNA samples from Malawi were chosen to undergo whole-genome sequencing to assess recent positive selection in OCM-related genomic regions. These samples were selected to have an approximately equal distribution of males and females and an equal number of ESAM and NESAM samples to minimize sampling bias. WGS was performed by first generating PCR-free libraries from one microgram genomic DNA using the TruSeq® DNA PCR-Free HT Sample Preparation Kit (Illumina, San Diego, CA, USA). The median insert sizes were approximately 400 bp. Libraries were tagged with unique dual-index DNA barcodes to allow the pooling of libraries and to minimize the impact of barcode hopping. Libraries were then pooled for sequencing on the NovaSeq 6000 (Illumina) to obtain at least 300 million 151-base read pairs per individual library. The sequence data were then aligned to human_g1k_v37_decoy.fasta via BWA-MEMv0.7.15^85^. GATK Resource Bundle B37^86^ was used to sort the data, mark duplicates, perform BQSR, and run the GATK Haplotype Caller to generate gVCF files on each sample. Then individual gVCFs were merged into a multi-sample VCF file using GATK 4.3.0.0. GATK Best Practice^87^ for variant calling and filtering was followed to obtain high-quality variant data, i.e., applying GATK VQSR function, followed by harder filters (filter parameters set as DP > = 10, QUAL > = 60, and ExcessHet <= 54.69).

We used the Integrated haplotype Score (iHS)^88^ to identify genomic signatures of selection in the Malawi dataset; this analysis is agnostic of phenotype and covariates. To compare signatures of selection at OCM loci between different countries, we created subsets of country-specific signatures of selection calculated by the H3Africa consortium^46^ using selscan^89^ with the same QC and run parameters as the Malawi dataset. This data included selection statistics for SNPs across OCM loci used in the association study: Benin (*n* = 45,537 SNP scores), Botswana (BOT, *n* = 44,277 SNP scores), Cameroon (CAM, *n* = 44,458 SNP scores), Mali (MAL, *n* = 43,033 SNP scores), Berom from Nigeria (BRN, *n* = 44,177 SNP scores), Gur speakers from West Africa (GWR, *n* = 43,523 SNP scores), and Zambia (ZAM, *n* = 47,572 SNP scores). These samples were sequenced on Illumina HiSeq 2500 and subsequently processed and mapped to GRCh37. SNPs with iHS scores >|2| were considered outliers, potentially under selection. Selection signatures were visualized using R^76^ and ggplot2^90^.

### Statistics & reproducibility

No statistical methods were used to predetermine sample size. Sample sizes were based on available cohorts and are consistent with prior studies of severe acute malnutrition. The primary analysis included 711 individuals after quality control (Jamaica, *n* = 340; Malawi, *n* = 371), with approximately balanced representation of ESAM and NESAM. Whole-genome sequencing analyses were performed in a subset of 151 individuals selected to balance sex and phenotype.

Genome-wide association analyses were performed using linear mixed models to account for relatedness and population structure, with sex and the leading multidimensional scaling component included as covariates. Association tests were two-sided. Bonferroni correction was applied to define genome-wide and locus-level significance thresholds, and test statistic inflation was assessed using quantile–quantile plots.

Cumulative association at one-carbon metabolism (OCM) loci was evaluated using permutation testing (10,000 iterations), comparing the observed proportion of significant SNPs to empirical null distributions derived from genome-wide and pathway-matched SNP sets. These tests were one-sided, and significance was defined as the proportion of permutations exceeding the observed value.

Mendelian randomization analyses were conducted using inverse-variance weighted and MR–Egger methods. Metabolite levels were log-transformed prior to analysis. No multiple testing correction was applied to MR analyses.

Selection analyses used integrated haplotype scores, with |iHS | > 2 considered suggestive of recent positive selection. Comparisons between groups were performed using two-sided tests.

All analyses were conducted using established software with standard parameters as described in the Methods. Key findings were supported across independent population groups and through sensitivity analyses.

### Reporting summary

Further information on research design is available in the Nature Portfolio Reporting Summary linked to this article.

## Supplementary information

Supplementary Information

Description of Additional Supplementary Files

Supplementary Data 1-8

Reporting Summary

Transparent Peer Review file

## Data Availability

The raw genome sequencing and genotyping data supporting the current study are subject to controlled access and have not been deposited in an unrestricted public repository because the ethical approvals and consent frameworks in Malawi and Jamaica accompanying recruitment and sample collection predated current data-sharing protocols and do not explicitly permit open public release of individual-level genomic data. Investigators seeking access to study-based data for research purposes should contact the corresponding author. Requests should include a brief research proposal, intended use of the data, evidence of appropriate institutional ethics approval or exemption, and a data security plan. Requests will be reviewed by the study investigators and, where appropriate, relevant institutional or local ethics/governance bodies. We aim to respond to data access requests within 8 weeks of receipt of a complete request. Approved access will require execution of a data use agreement specifying that data may be used only for the approved research purpose, may not be redistributed or used to attempt participant re-identification, and must be handled in accordance with applicable institutional, national, and international requirements for human genomic data. URLs for online data, information, software, and resources used or generated in this study are provided below: • H3Africa array v1.1 - https://h3africa.org/index.php/2019/12/12/h3africa-chip-faq/. • Infinium H3Africa Consortium Array - https://www.illumina.com/content/infinium-h3africa-consortium-array-data-sheet. • H3Africa/H3ABioNet Imputation Service. H3ABioNet project. Accessed on **2 April 2019**. Available at: https://www.h3abionet.org/resources/h3abionet-imputation-service. • Michigan Imputation Server—https://imputationserver.sph.umich.edu/#!. • 1000 Genomes Project Resource - https://www.internationalgenome.org/. • KwashNet - https://www.genome.gov/research-at-nhgri/projects/KwashNet. • QGIS Association - http://www.qgis.org.

## Code availability

The code used in this study can be found at GitHub: SAM_OCMgenetics [https://github.com/NHGRI/SAM_OCMgenetics], and Zenodo (https://doi.org/10.5281/zenodo.20632614) [https://zenodo.org/records/20632614].
