# Supplementary material for: Aberrant one-carbon metabolism and ancestral genetics underlie edematous severe acute malnutrition
Source: Nat Commun. 2026 Oct 15;17:10798. doi: 10.1038/s41467-026-74239-x (PMC13669358; doi:10.1038/s41467-026-74239-x)
Supplement: Supplementary file 1 [file 41467_2026_74239_MOESM1_ESM.pdf]

Supplementary Figures

Aberrant One-Carbon Metabolism and Ancestral Genetics Underlie Edematous Severe Acute Malnutrition

(Lie et. al.)

Supplementary Figure 1 – Data analysis flowchart

Supplementary Figure 2 – 2D plots of Multidimensional scaling (MDS) components 1-3

Supplementary Figure 3 – Scree plots of Proportion of Variance attributable

Supplementary Figure 4 – LocusZoom plots of top OCM loci

Supplementary Figure 5 – Minor allele frequencies and effect sizes in Jamaica and Malawi

Supplementary Figure 6 – Haploview and linkage disequilibrium (LD) plots for associated SNPs at *GABBR2* in Jamaica and Malawi

Supplementary Figure 7 – Admixture plots for African populations

Supplementary Figure 8 – Violin plots of individual ancestry proportions

Supplementary Figure 9 - Proportion of SNPs at OCM loci with absolute Standardized iHS score > 2

Supplementary Figure 10 – Density plots of standardized iHS scores by SAM category

Supplementary Figure 11 – Manhattan and QQ plot of genome-wide association

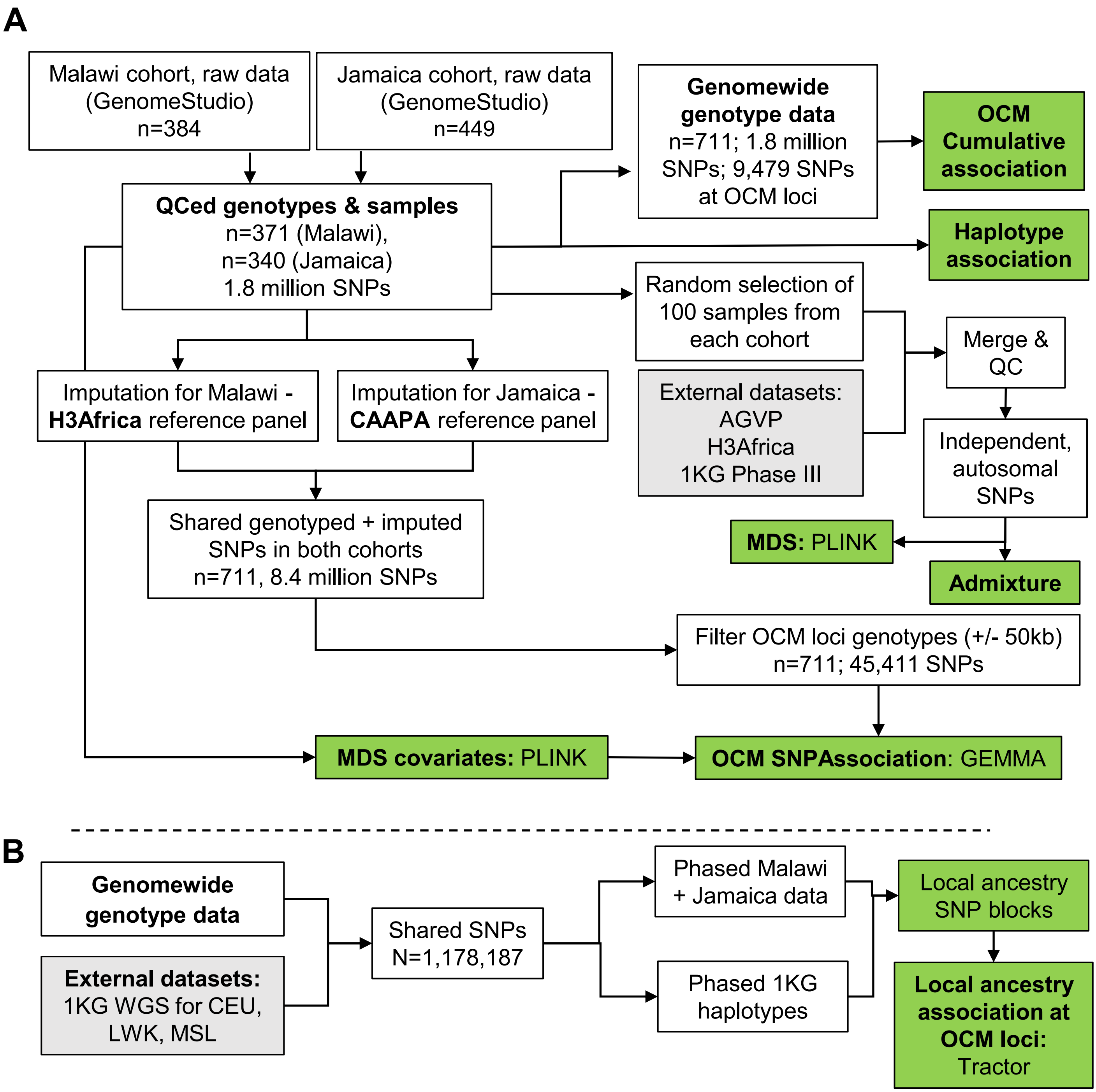

Supplementary Fig. 1 – Flowchart of SAM-OCM analyses. S1A – Data processing and analysis steps for genetic association tests and admixture analysis. **S1B** – Data processing and analysis steps for ancestry-adjusted association analysis. Boxes labelled as "Pooled genotype data" in S1A and S1B refer to the same set of genetic data. 1KG – 1000 Genomes Project populations; MDS – Multidimensional Scaling; QC – Quality control, which includes SNP and sample missingness, gender checks, Hardy-Wienberg equilibrium, and familial relatedness. CAAPA – Consoritum on Asthma among African ancestry Populations in the Americas; H3Africa – Human Health and Heredity in Africa Consortium; AGVP – African Genome Variation Project; 1KG – 1000 Genomes Project

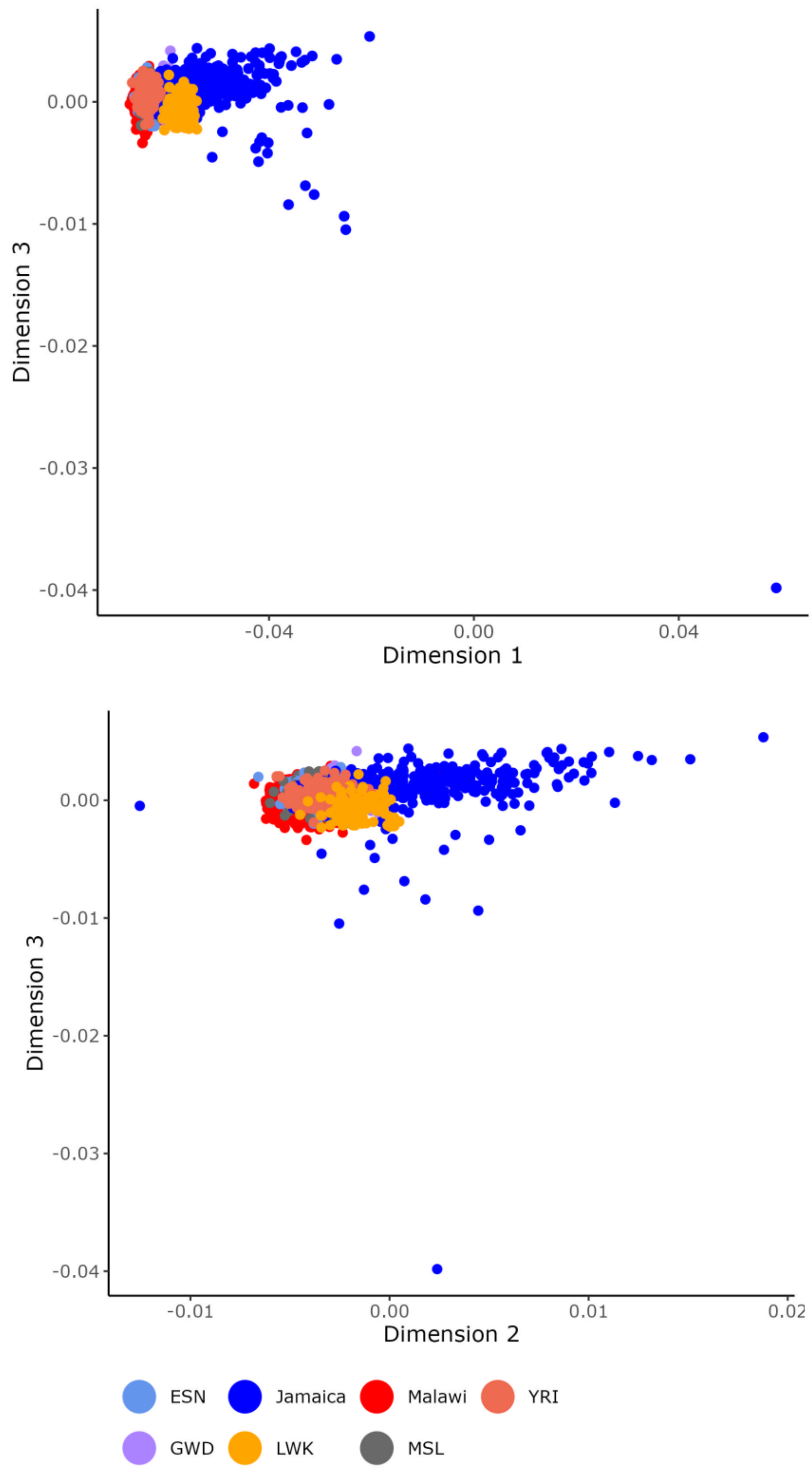

Supplementary Fig. 2 – 2D plots of Multidimensional scaling (MDS) Dimensions 1 to 3. Population abbreviations are per the 1000 Genomes Project: ESN – Esan in Nigeria; GWD – Gambians from Western Division; LWK – Luyha in Webuye, Kenya; MSL - Mende in Sierra Leone; YRI – Yoruba in Ibadan, Nigeria

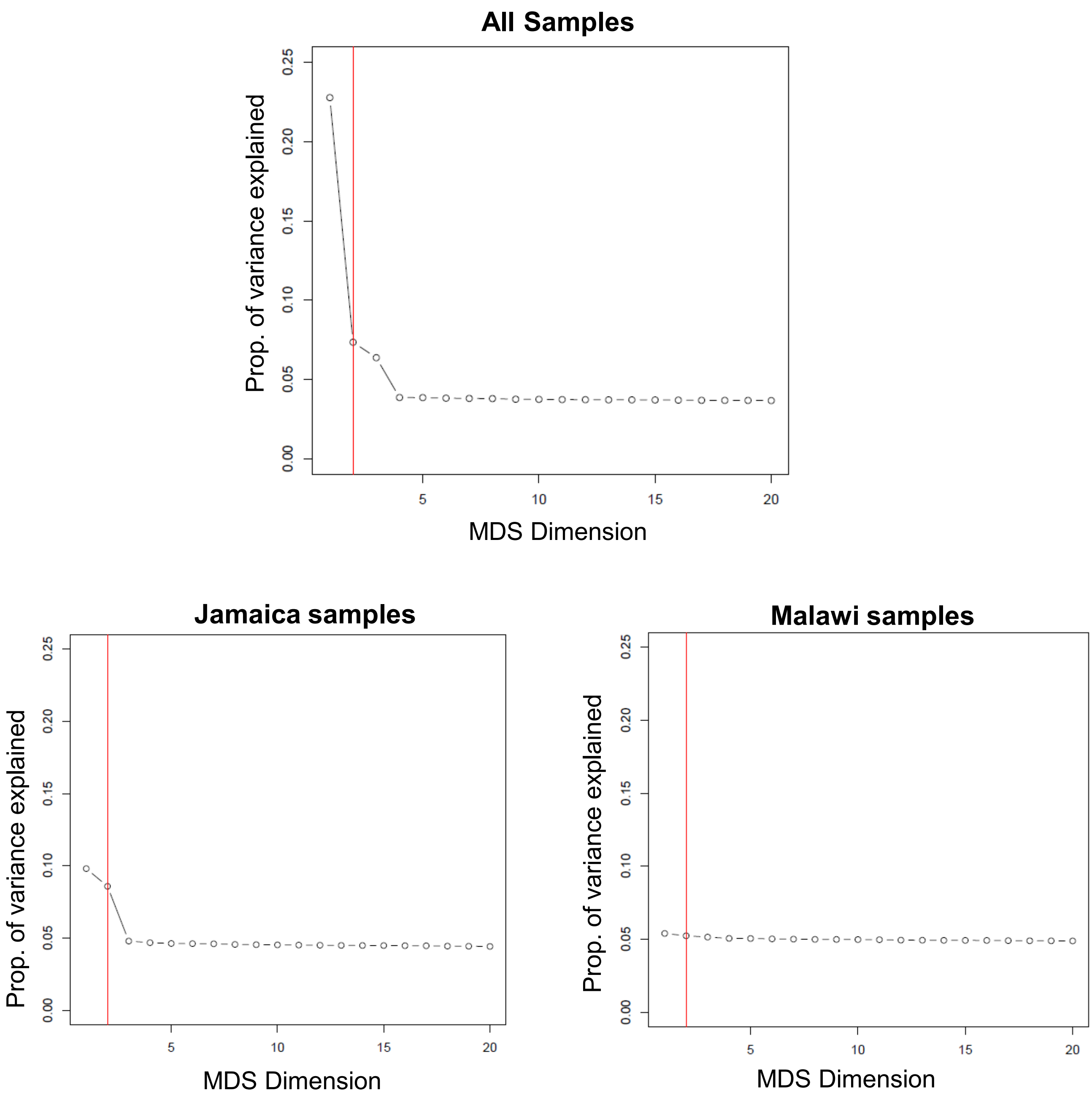

Supplementary Fig. 3 – Multidimensional Scaling (MDS) variance scree plots - MDS eigenvalues are calculated based on first 20 MDS dimensions and scree plots were generated for three cohorts – All samples, Jamaica samples alone and Malawi samples alone.

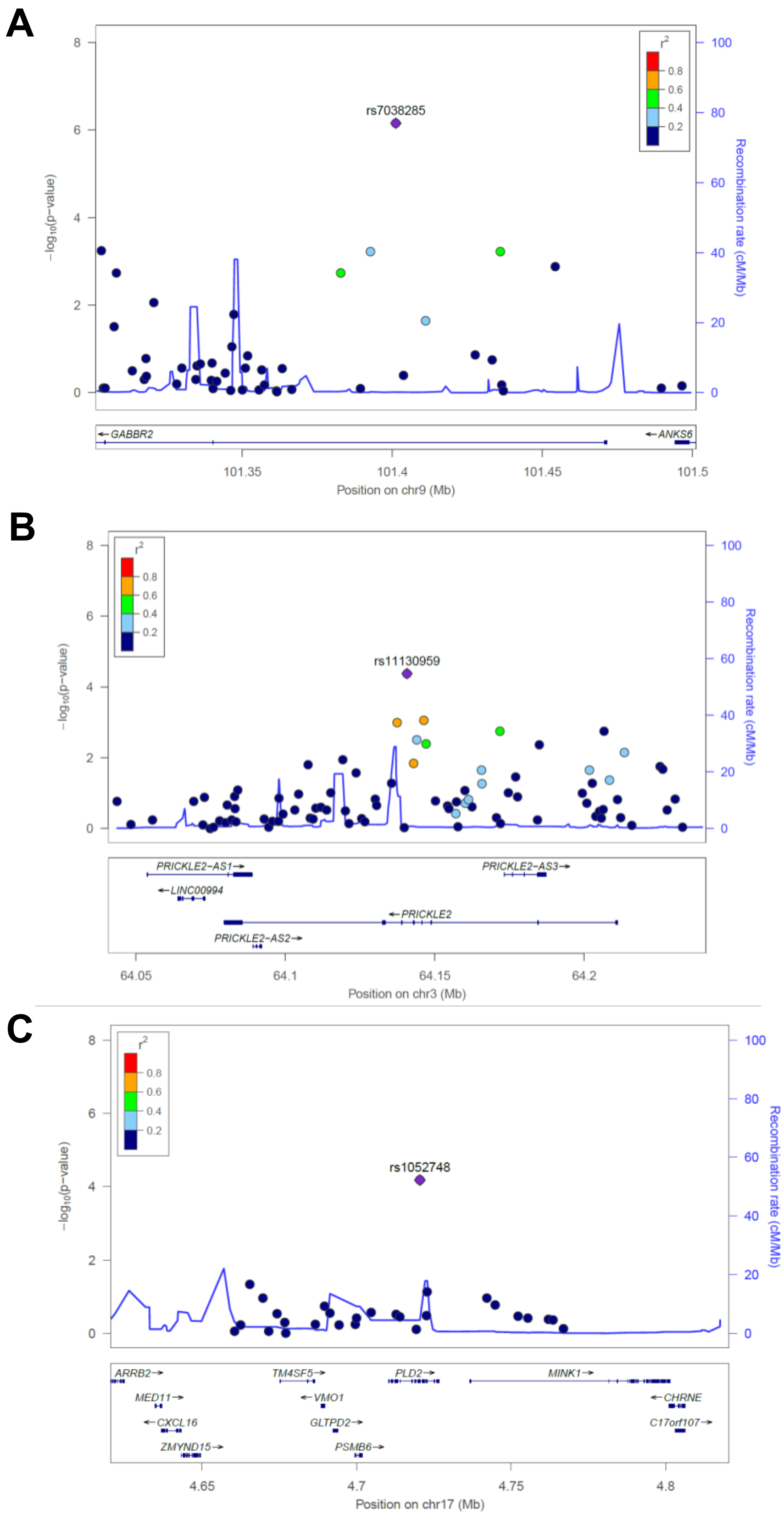

Supplementary Fig. 4 – LocusZoom plots of top OCM loci associated with ESAM. S1A – *GABBR2* on chromosome 9; S1B- *PRICKLE2* on chromosome 13; S1C- *PLD2* on chromosome 17. Recombination rates and LD (r^2) are given relative to 1000 AFR populations

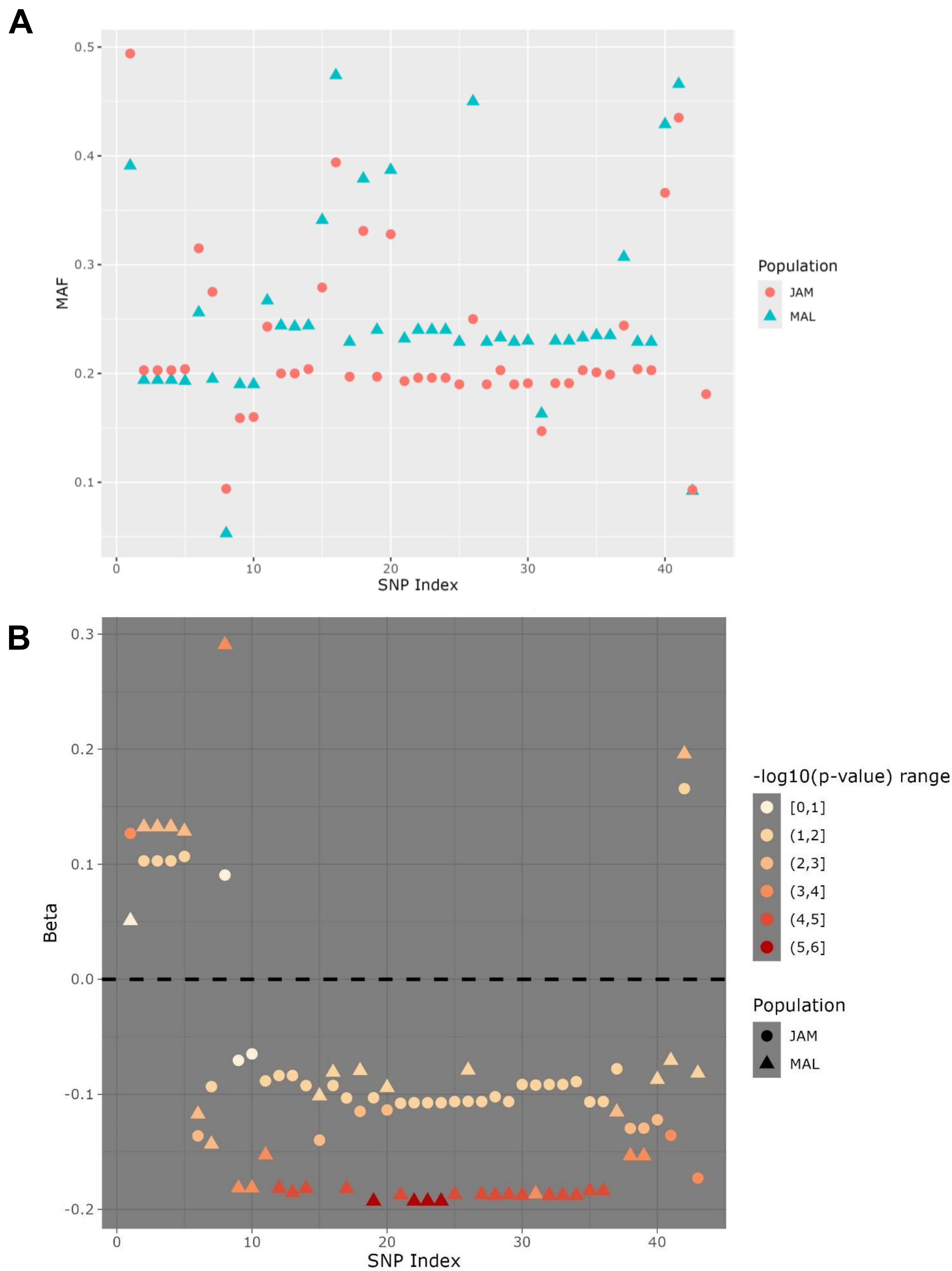

Supplementary Fig. 5– Minor allele frequencies (MAF; **S5A**) and effect sizes (Beta; **S5B**) in Jamaica and Malawi for ESAM associated OCM SNPs indexed in Supplementary Tables S3 & S4.

A

REF	G C A A G
ALT	A A G G A

GABBR2

	MAF_Jam	MAF_Mal	P_Jam	P_Mal
A A G G A	0.141	0.163	0.049	7.575E-04
A C A A A	0.085	0.104	0.072	0.551
G C A A A	0.150	0.141	0.774	0.304
G C A A G	0.562	0.509	8.307E-04	0.116

B

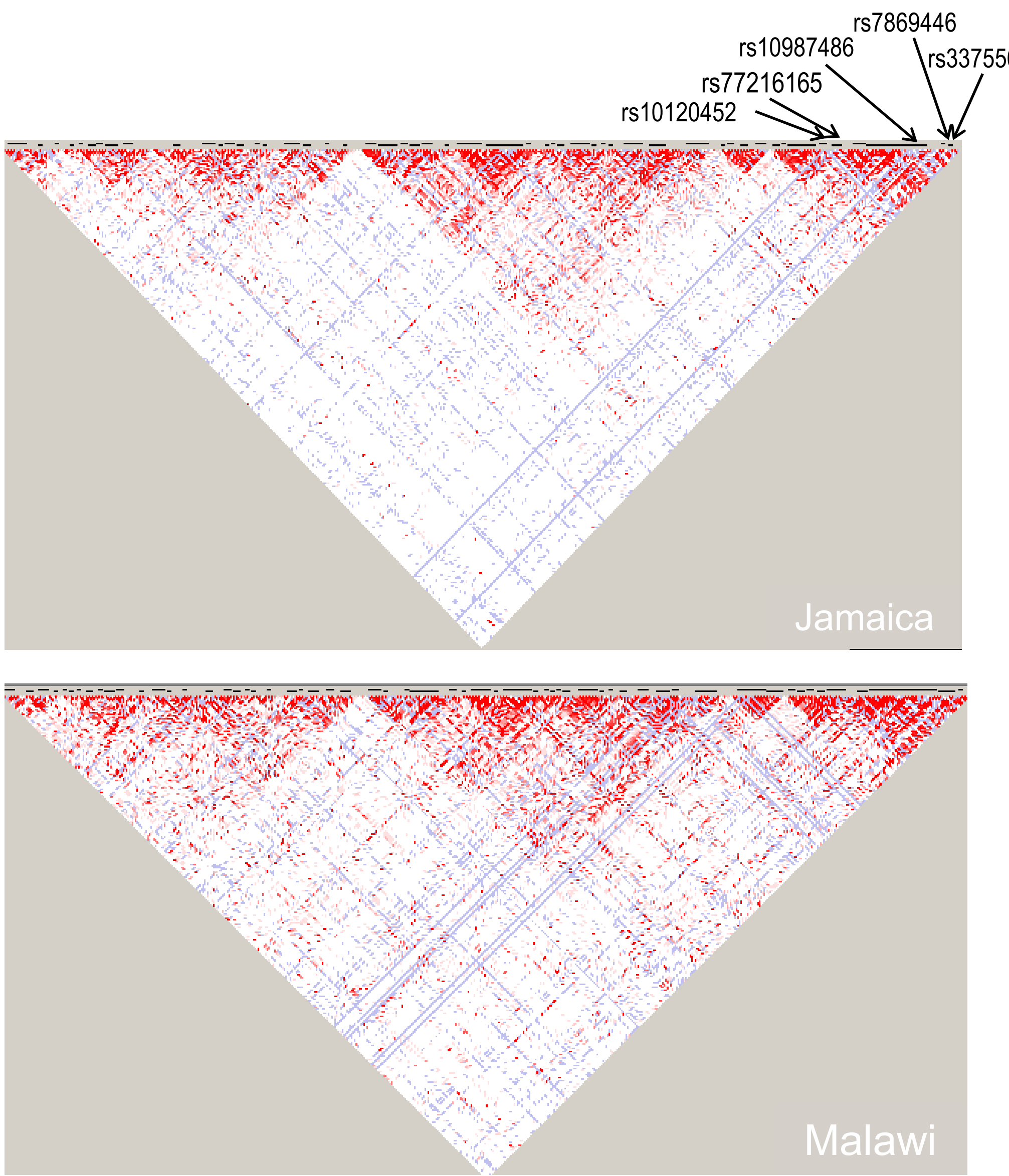

Supplementary Fig. 6– Haplotype association (A) and linkage disequilibrium (LD) (B) for SNPs surpassing locus threshold at *GABBR2* in Jamaica and Malawi. Haplotype SNPs [rs10120452; rs77216165; rs10987486; rs7869446; rs337556] are as ordered from 5-prime to 3-prime. MAF – minor allele frequency. In the LD plot, SNPs are plotted on horizontal and color at intersection of two SNPs represents pairwise LD (D'), where red = D' >0.9; blue D' 0.6-0.89; white D' <0.6.

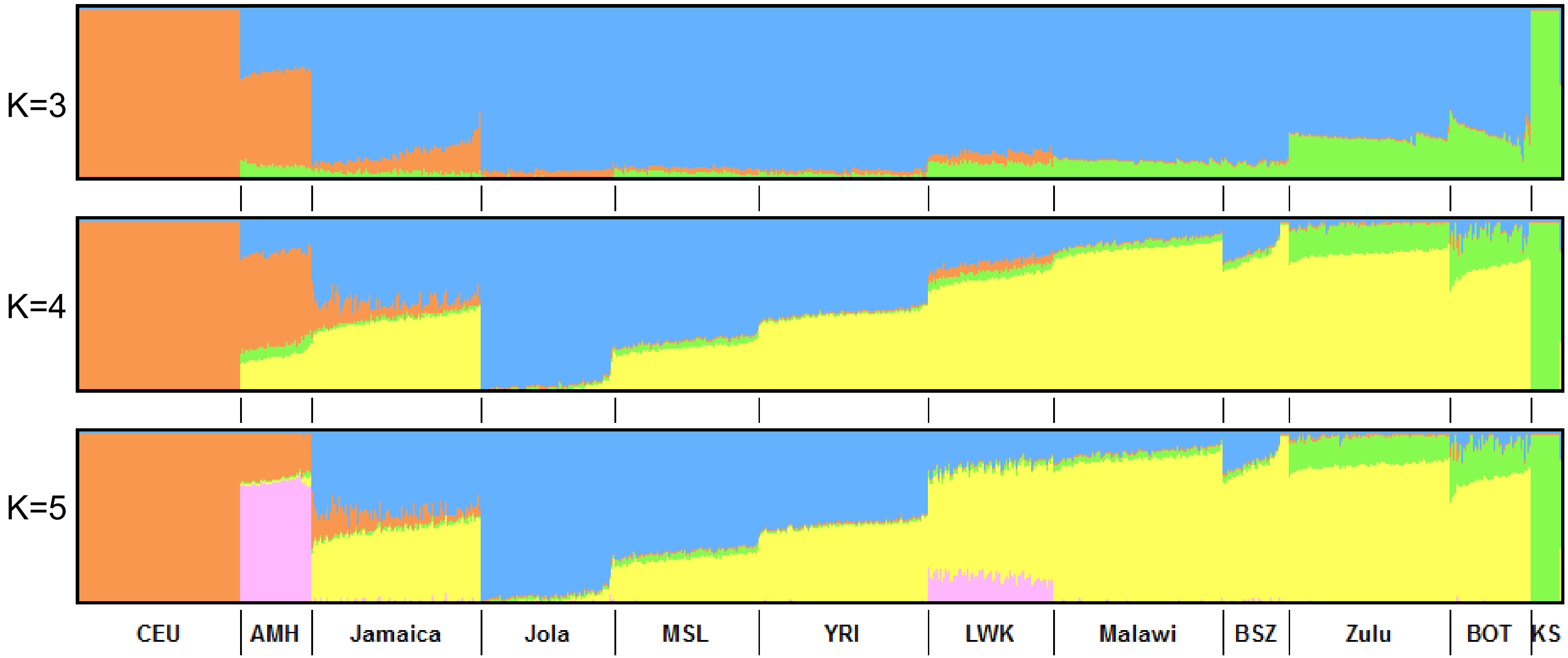

Supplementary Fig. 7– Admixture plots for Jamaica and Malawi alongside potential ancestral source populations. Country codes are as given in Figure 4b. The CV error is minimized at K=5.

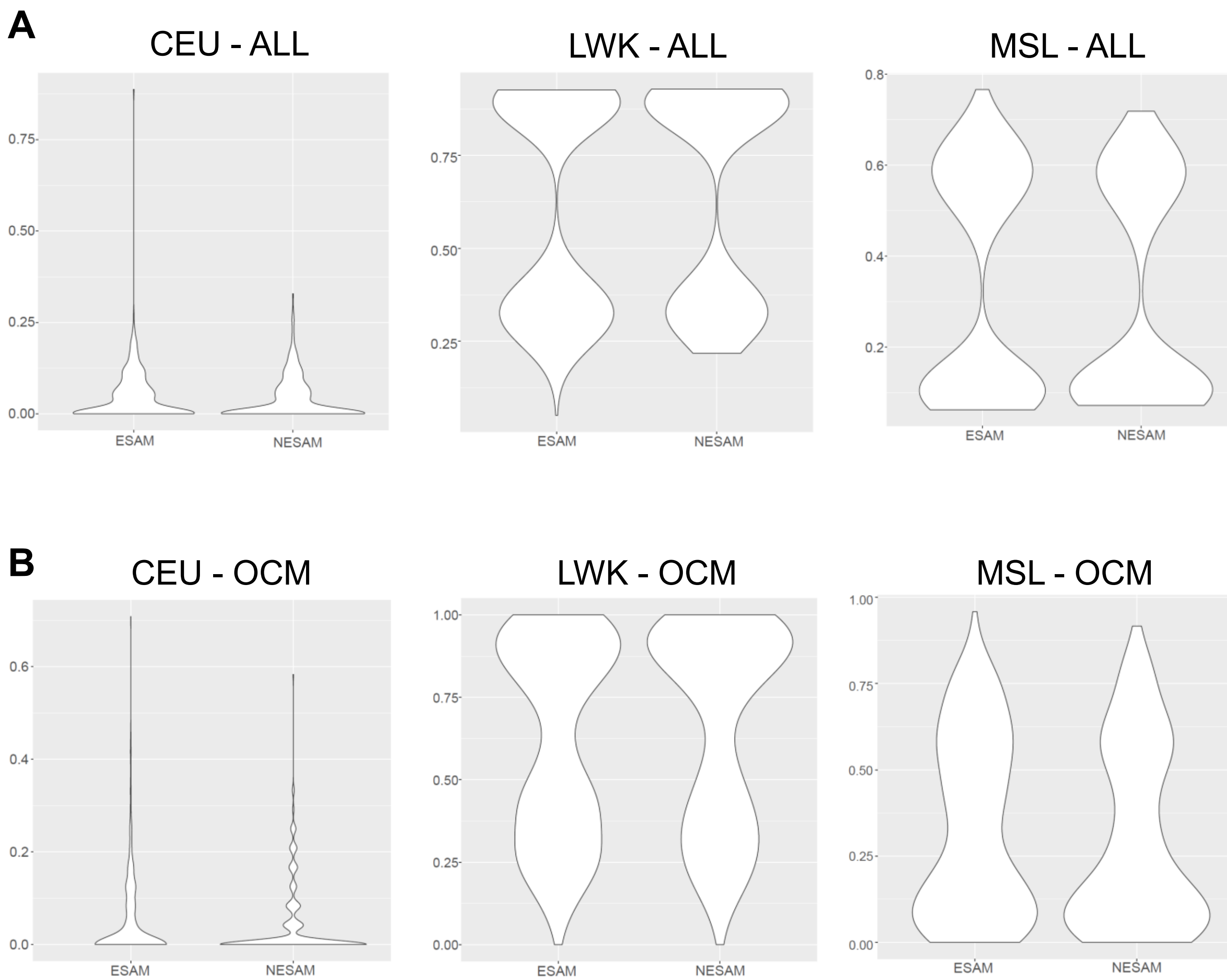

Supplementary Fig. 8– Violin plots of individual ancestry proportions in ESAM and NESAM across all SNPs (**A**) and at OCM SNPs (**B**). CEU = CEPH from Utah; LWK = Luyha from Weyube, Kenya, MLS = Mende from Sierra Leone.

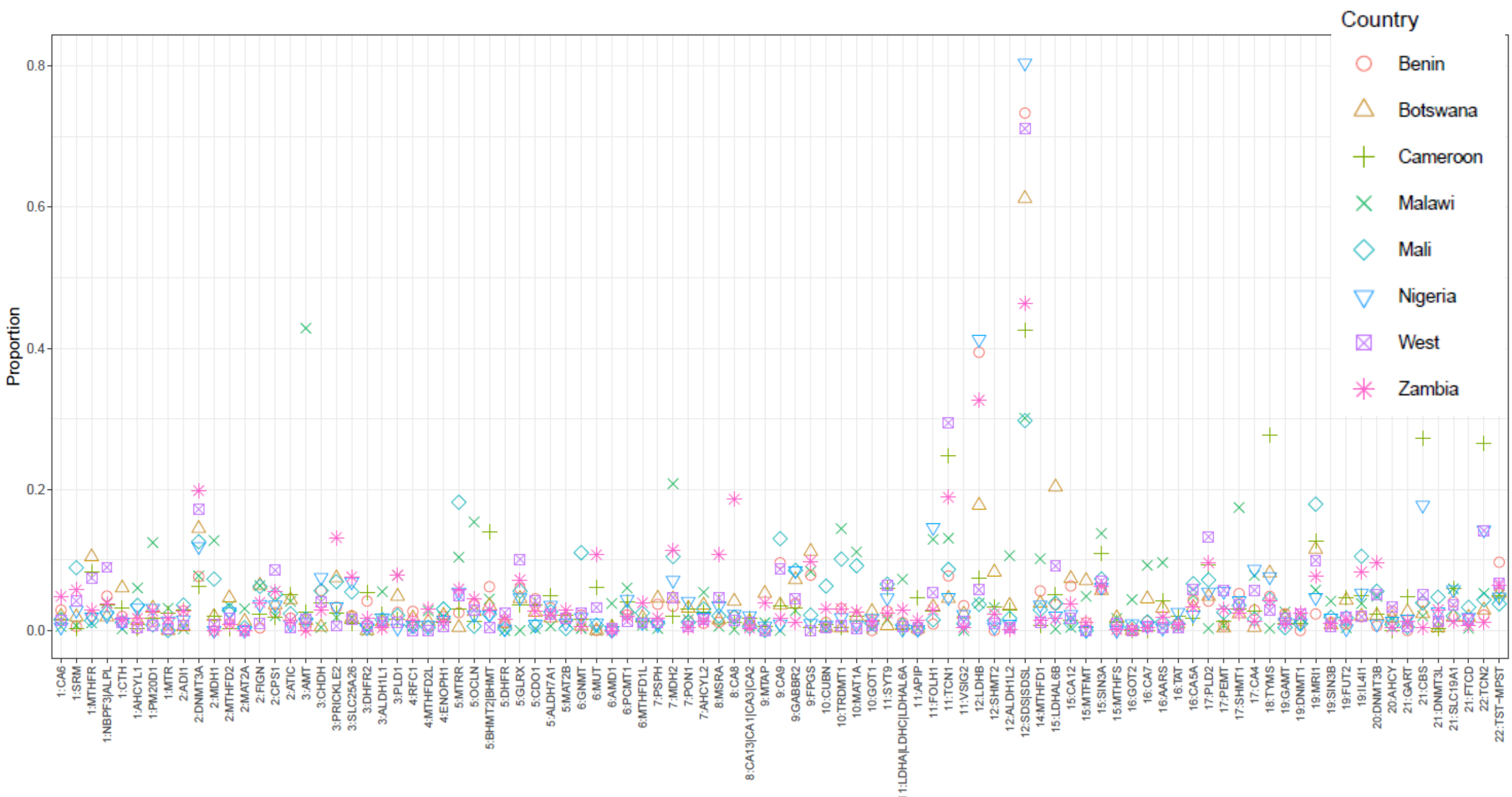

Supplementary Fig. 9– Proportion of SNPs at OCM loci with absolute standardized iHS scores > 2 in H3Africa populations and among Malawi WGS cohort.

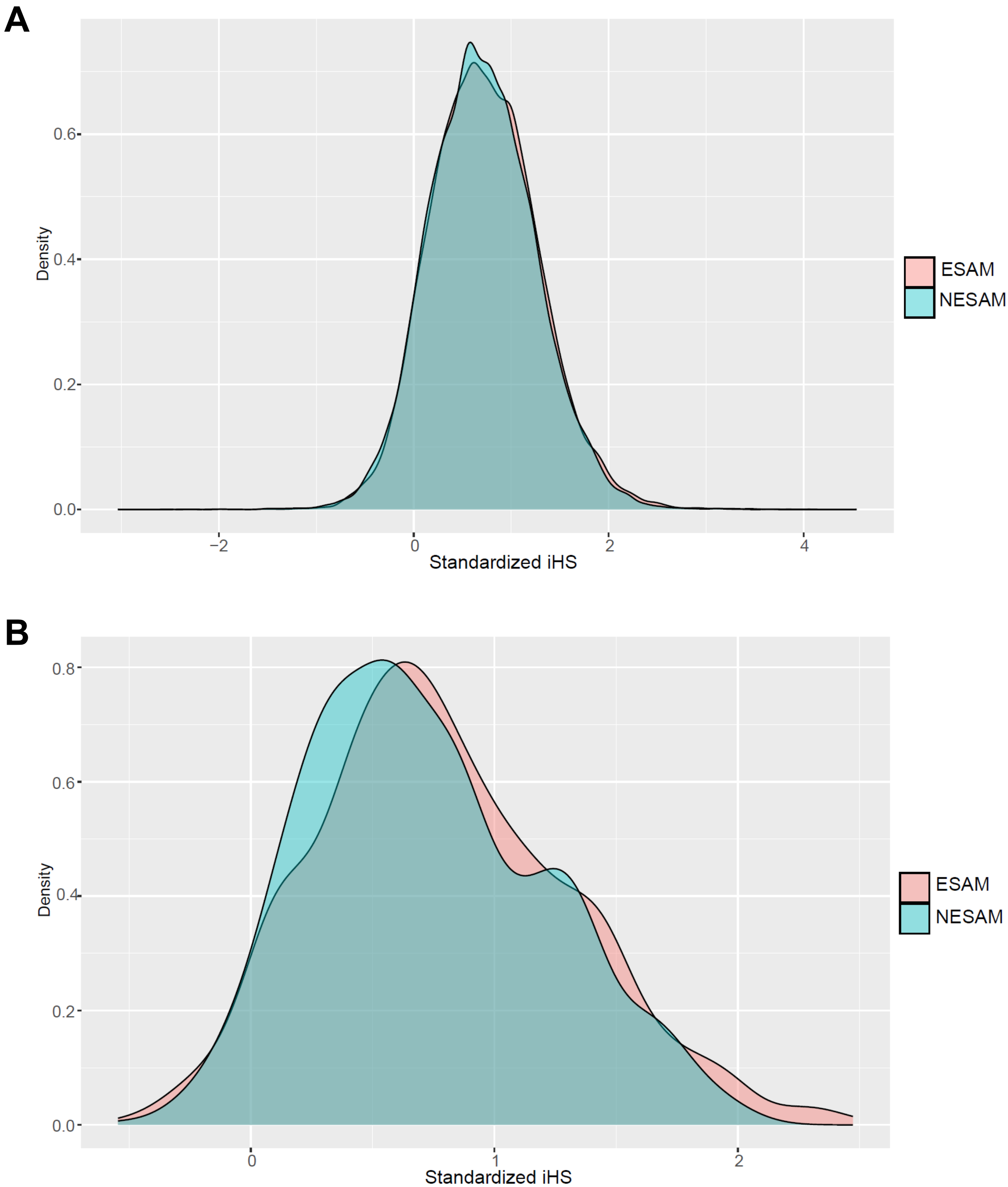

Supplementary Fig. 10– Density plots of Standardized iHS scores by SAM category for all SNPs genomewide (**A**) and across OCM loci (**B**).

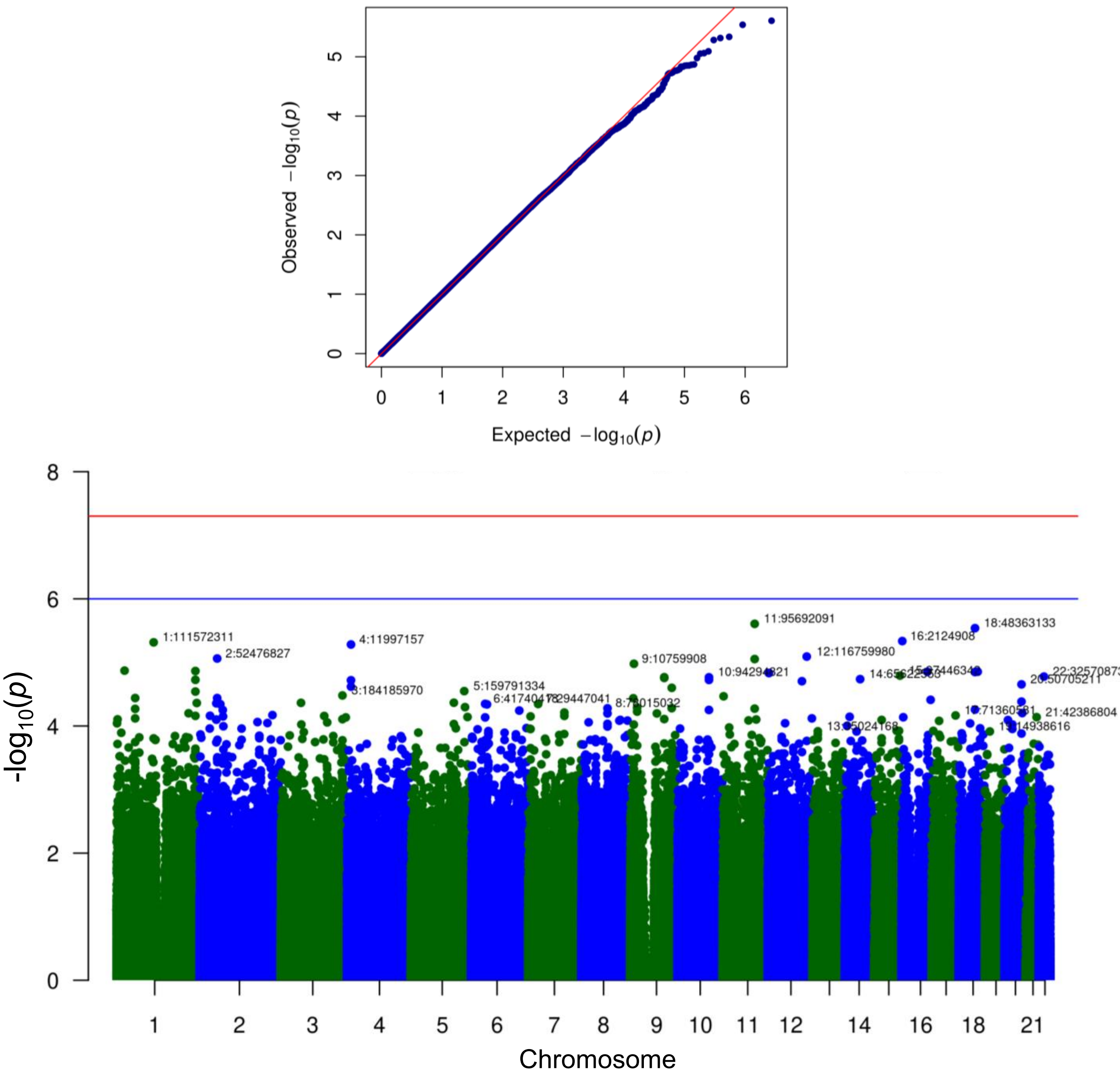

Supplementary Fig. 11 – Manhattan and Quantile-Quantile plots of genome-wide association for ESAM using SNPs with minor allele frequency above 5% and covariates of sex and components 1-3 of multidimensional scaling.

Supplementary Methods

Aberrant One-Carbon Metabolism and Ancestral Genetics Underlie Edematous Severe Acute Malnutrition

(Lie et. al.)

SNP Genotyping and Validation in cell free DNA

Six target SNPs were targeted for genotyping based on their relative P-value (at or below loci-level significance) and the suitability of the surrounding sequence for sequencing (i.e. the ease of primer creation). The tested SNPs were genotyped from cell free DNA using complementary methodologies.

- **Sanger sequencing (SeqStudio)** was employed for genotyping rs753562, rs17664202, rs77216165, rs7869446, and rs651495.
- **Pyrosequencing (PyroMark Q48 Autoprep)** was utilized for genotyping rs1052748.

Primers for each SNP are given in Supplementary Table 1 and are reproduced below.

SNP location	rsID	Gene	PCR Primer	PCR Primer Seq
chr3:64137371	rs753562	*PRICKLE2*	chr3:64137371- FOR	CTCTGCCAAGTACTGAGCAAAAAT
			chr3:64137371- REV	ACAGCCTGTTGCTTCAACTCA
chr3:64146322	rs17664202	*PRICKLE2*	chr3:64146322 - FOR	TGTGCCCATGCAAGTGATCC
			chr3:64146322- REV	GCACAATTCCCAAGGTGTGCATAA
chr9:101414156	rs77216165	*GABBR2*	chr9:101414156- FOR	AGGCTGAGGAAGGGTTGAAG
			chr9:101414156- REV	ACAGACTATCCTCCCAAATCACT
chr9:101462262	rs7869446	*GABBR2*	chr9:101462262-- FOR	GCATCCCTAGAAAGCCAGGTA
			chr9:101462262- REV	CACTGTCCAACAGGTGATCAAG
chr17:18311534	rs651495	*SHMT1*	chr17:18311534- FOR	ATGCAAGCACTGAACACTGG
			chr17:18311534- REV	ATGGAAATTCAAACGGGCCC
chr17:4720469	rs1052748	*PLD2*	OCM_SNP_chr17_4720469_FOR	CAGACCACCAAGGCCAAGTACA
			OCM_SNP_chr17_4720469_REV (Biotin tagged)	CTGCCCTCCTGGAAGTGTGAA

Highlighted SNPs – assay unsuccessful; not used in analyses

A. Validation via Dideoxy (Sanger) Sequencing

Sanger sequencing was performed using the SeqStudio Genetic Analyzer to validate selected single nucleotide polymorphisms (SNPs). The workflow included primer design, PCR amplification, cycle sequencing, purification, capillary electrophoresis and analysis.

Primer Design and PCR Amplification

M13-tagged primers targeting SNP regions were designed using the UCSC *in silico* primer design tool, based on the GRCh38/hg38 genome assembly. Primer specificity was confirmed via BLAT alignment, and primers were synthesized by IDT. Stock solutions were prepared at 100 µM in TE buffer.

PCR Reaction Mix:

Component	Volume
Genomic DNA (4 ng/µL)	1.0 µL
M13-tailed PCR primer mix (0.8 µM each primer)	1.5 µL
BigDye® Direct PCR Master Mix	5.0 µL
Deionized water	2.5 µL

Total volume for each reaction	10.0 µL

Thermocycling conditions:

Stage	Temp	Time
Hold	95°C	10 min
Cycle (35 cycles)	96°C	3 sec
	62°C	15 sec
	68°C	30 sec
Hold	72°C	2 min
Hold	4°C	

PCR amplification was confirmed via 2% agarose gel electrophoresis.

Cycle Sequencing:

Forward and reverse sequencing reactions were prepared using BigDye® Direct Sequencing Master Mix with M13-tagged primers. The reaction components were as follows:

Component	Volume (per reaction)
BigDye® Direct Sequencing Master Mix	2.0 µL
M13 Forward or Reverse Primer	1.0 µL
**Total Volume**	3.0 µL

The sequencing reaction was set up on ice and processed in a thermal cycler under the following conditions:

Stage	Veriti® thermal cyclers		9700 thermal cycler	
	Temp	Time	Temp	Time
Hold	37°C	15 min	37°C	15 min
Hold	80°C	2 min	80°C	2 min
Hold	96°C	1 min	96°C	1 min
Cycle (25 cycles)	96°C	10 sec	96°C	10 sec
	50°C	5 sec	50°C	5 sec
	60°C	75 sec	60°C	4 min
Hold	4°C	∞	4°C	∞

Purification and Capillary Electrophoresis:

Sequencing products were purified using the BigDye XTerminator® Purification Kit. A premix of SAM™ Solution and XTerminator® Solution was prepared and vortexed to homogeneity. Samples were incubated at 24°C for 20 minutes with mixing at 2000 rpm, followed by centrifugation at 1000×g for 2 minutes.

Capillary electrophoresis was performed on the SeqStudio Genetic Analyzer. Run conditions were optimized using the "Z_BigDye_Direct" dye set. Sample names and plate configurations were

assigned using Plate Manager software. Sequencing runs were initiated following system calibration and buffer replacement.

Data Analysis:

Following sequencing, *.ab1* files were exported and analyzed using Sequencher software. Primer reference sequences were used for alignment, and SNPs were identified based on sequence variations. Genotypes were assigned through comparative analysis with the reference genome.

B. Validation of SNP via Pyrosequencing on PyroMark Q48 Autoprep

Overview:
Genotyping for rs1052748 (chr17:4720469, gene *PLD2)* was performed using the PyroMark Q48 Autoprep system, which employs Pyrosequencing® technology for precise sequence-based variant detection. This approach enables rapid and quantitative genotyping, making it well-suited for genetic analyses.

Primer Design for Genotyping:
Biotinylated primers for SNP genotyping were designed using PyroMark Assay Design SW 2.0. The target sequence was input into the software, and the dispensation order was generated. The assay parameters were finalized by locking the design, and the assay file (*.qsnp*) was saved for subsequent sequencing. Biotinylated primer sets were procured through Qiagen's GeneGlobe platform.

PCR Amplification

PCR Reaction Mix:

Component	Volume per Reaction
PyroMark PCR Master Mix	12.5 µL
Cora Load	2.5 µL
Q Solution	5 µL
Primer Mix	2.5 µL
DNA (10ng/ul)	2.5ul

Optimized PCR Thermocycling Conditions:

Step	Temperature	Duration	Cycles
Initial Denaturation	95°C	15 minutes	1
Denaturation	95°C	30 seconds	40
Annealing	55-65°C	30 seconds	40
Extension	72°C	30 seconds	40
Final Extension	72°C	10 minutes	1

Amplification was confirmed via 2% agarose gel electrophoresis.

Preparation of Pyrosequencer Cartridges: Prior to use, Pyrosequencer cartridges were cleaned according to the manufacturer's instructions. Primers and reagents were manually loaded in the designated cartridges.

PyroMark Disc Preparation: A PyroMark Q48 Disc was placed into the PyroMark Q48 Autoprep instrument. Each designated well was loaded with 3 µL of PyroMark Q48 Magnetic Beads and 10 µL of biotinylated PCR product. Sample placement was verified using the PyroMark Q48 Software, ensuring correct loading. The instrument lid was secured, and the bead immobilization process was initiated prior to sequencing.

Assay Setup in PyroMark Q48 Autoprep Software: In the PyroMark Q48 Autoprep Software, assays were selected from the toolbar, and the target sequence was input. The dispensation order was generated and locked to finalize assay parameters, and the assay file (*.qsnp*) was saved.

Run Setup and Execution: A new run setup was created by assigning a run name and saving the configuration. Assays were loaded into designated wells, with optional assignment of sample IDs. The standard sequencing protocol was selected. The finalized setup was saved as a *.qrun* file for execution.

Data Analysis: Upon completion of the sequencing run, the *.qrun* file was transferred to a computer equipped with PyroMark Q48 Autoprep Software for analysis. The SNP analysis mode was selected from the toolbar to facilitate genotyping of single nucleotide polymorphisms. Resulting data were visualized in the Pyrogram, where genotype calls (e.g., homozygous or heterozygous) were determined based on peak patterns.

Supplementary Data:

Supplementary Data 1 - Clinical and demographic characteristics of Malawi and Jamaica cohorts

Supplementary Data 2 - One Carbon Metabolism Loci evaluated

Supplementary Data 3 - Association statistics at loci surpassing the pre-defined locus-level threshold for association

Supplementary Data 4 - ESAM-associated SNPs successfully genotyped from cell-free DNA using PCR amplification

Supplementary Data 5 - Association statistics and minor allele frequency (MAF), Odds Ratio (OR) and associated P values for SNPs at OCM loci in Jamaican and Malawian cohorts

Supplementary Data 6 - proportion of SNPs at OCM loci with |iHS scores| > 2 in Malawi WGS cohort and H3Africa populations.

Supplementary Data 7 - iHS scores for available ESAM-associated SNPs at OCM loci in Malawi WGS cohort and H3Africa populations.

Supplementary Data 8 - Genomewide (non-OCM) SNPs surpassing 1E-04 in genome-wide analyses.
