# Supplementary material for: Aberrant one-carbon metabolism and ancestral genetics underlie edematous severe acute malnutrition
Source: Nat Commun. 2026 Oct 15;17:10798. doi: 10.1038/s41467-026-74239-x (PMC13669358; doi:10.1038/s41467-026-74239-x)
Supplement: Supplementary file 2 [file 41467_2026_74239_MOESM2_ESM.pdf]

Description of Additional Supplementary Files

Supplementary Data 1 - Clinical and demographic characteristics of Malawi and Jamaica cohorts

Supplementary Data 2 - One Carbon Metabolism Loci evaluated

Supplementary Data 3 - Association statistics at loci surpassing the pre-defined locus-level threshold for association

Supplementary Data 4 - ESAM-associated SNPs successfully genotyped from cell-free DNA using PCR amplification

Supplementary Data 5 - Association statistics and minor allele frequiency (MAF), Odds Ratio (OR) and associated P values for SNPs at OCM loci in Jamaican and Malawian cohorts

Supplementary Data 6 - proportion of SNPs at OCM loci with |iHS scores| > 2 in Malawi WGS cohort and H3Africa populations.

Supplementary Data 7 - iHS scores for available ESAM-associated SNPs at OCM loci in Malawi WGS cohort and H3Africa populations.

Supplementary Data 8 - Genomewide (non-OCM) SNPs surpassing 1E-04 in genome-wide analyses.
