# Supplementary material for: Aberrant one-carbon metabolism and ancestral genetics underlie edematous severe acute malnutrition
Source: Nat Commun. 2026 Oct 15;17:10798. doi: 10.1038/s41467-026-74239-x (PMC13669358; doi:10.1038/s41467-026-74239-x)
Supplement: Supplementary file 3 [file 41467_2026_74239_MOESM4_ESM.pdf]

nature portfolio

Corresponding author(s): Neil Hanchard

Last updated by author(s): Jun 26, 2025

Reporting Summary

Nature Portfolio wishes to improve the reproducibility of the work that we publish. This form provides structure for consistency and transparency in reporting. For further information on Nature Portfolio policies, see our Editorial Policies and the Editorial Policy Checklist.

Statistics

For all statistical analyses, confirm that the following items are present in the figure legend, table legend, main text, or Methods section.

n/a	Confirmed	
☐	☒	The exact sample size (*n*) for each experimental group/condition, given as a discrete number and unit of measurement
☐	☒	A statement on whether measurements were taken from distinct samples or whether the same sample was measured repeatedly
☐	☒	The statistical test(s) used AND whether they are one- or two-sided<br>*Only common tests should be described solely by name; describe more complex techniques in the Methods section.*
☐	☒	A description of all covariates tested
☐	☒	A description of any assumptions or corrections, such as tests of normality and adjustment for multiple comparisons
☐	☒	A full description of the statistical parameters including central tendency (e.g. means) or other basic estimates (e.g. regression coefficient) AND variation (e.g. standard deviation) or associated estimates of uncertainty (e.g. confidence intervals)
☐	☒	For null hypothesis testing, the test statistic (e.g. F, t, r) with confidence intervals, effect sizes, degrees of freedom and P value noted<br>*Give P values as exact values whenever suitable.*
☒	☐	For Bayesian analysis, information on the choice of priors and Markov chain Monte Carlo settings
☒	☐	For hierarchical and complex designs, identification of the appropriate level for tests and full reporting of outcomes
☐	☒	Estimates of effect sizes (e.g. Cohen's d, Pearson's r), indicating how they were calculated

Our web collection on statistics for biologists contains articles on many of the points above.

Software and code

Policy information about availability of computer code

Data collection	GenomeStudio (Illumina, San Diego, California, USA)<br>Sequencher (software version 4.10.1)
Data analysis	BWA-MEMv0.7.15; GATK 4.3.0.0; Eagle v2.4; Selscan 2.0; PLINK v1.07; GEMMA v0.98.5; TRACTOR v1.1.0; ADMIXTURE v1.3; R v4.3

For manuscripts utilizing custom algorithms or software that are central to the research but not yet described in published literature, software must be made available to editors and reviewers. We strongly encourage code deposition in a community repository (e.g. GitHub). See the Nature Portfolio guidelines for submitting code & software for further information.

Data

Policy information about availability of data

All manuscripts must include a data availability statement. This statement should provide the following information, where applicable:

- Accession codes, unique identifiers, or web links for publicly available datasets
- A description of any restrictions on data availability
- For clinical datasets or third party data, please ensure that the statement adheres to our policy

The raw sequencing and genotyping data supporting the current study have not been deposited in a public repository, as Ethical approvals in Malawi and Jamaica accompanying recruitment and sample collection for this study predated current data sharing protocols and do not explicitly include publicly sharing data; however, investigators interested in study-based data access should directly contact the corresponding author.

Research involving human participants, their data, or biological material

Policy information about studies with human participants or human data. See also policy information about sex, gender (identity/presentation), and sexual orientation and race, ethnicity and racism.

Reporting on sex and gender	We have included both males and females (by both sex-at-birth and genetic sex), throughout our study.
Reporting on race, ethnicity, or other socially relevant groupings	Our study incorporates genetic ancestry as a biological variable to account for population stratification and potential ancestry-related genetic variation that may influence our outcomes. Genetic ancestry was determined through pathway-wide SNPs rather than through self-reported race or ethnicity categories. We utilized genetic ancestry rather than racial or ethnic categories because our research questions focus on biological mechanisms that may vary based on genetic background.
Population characteristics	The study cohort was recruited through major nutritional rehabilitation sites in Jamaica (in the Caribbean) and the African nation of Malawi. Both countries self-describe their populations as being of predominant African ancestry. All included participants will have had a clinical diagnosis of Severe Acute Malnutrition (SAM) in their medical history. SAM occurs in both sexes. We included sex, genetic information, and geographic recruitment site as covariates; no other population characteristics were included in the model building. Additional population details are available in PMID: 31857576.
Recruitment	All recruitment consisted of samples collected after individuals had received a diagnosis of either ESAM or NESAM. Samples from Jamaica included DNA from blood and buccal samples from children diagnosed with SAM. In Malawi, samples from children with SAM were recruited from 18 rural sites across five districts. Full details are given in PMID: 31857576.
Ethics oversight	In Jamaica, the study was approved by the Ethics Committees of the UHWI/University of the West Indies Faculty of Medical Sciences. In Malawi, the study was approved by the National Health Science Review Committee (NHSRC) of the Ministry of Health, Government of Malawi. In both countries, written informed consent was obtained from adult participants and the parent or adult guardian of participating children and all ethical regulations regarding human participants were complied with. Permission to use the participant samples for genetic studies was approved by the Institutional Review Board (IRB) of Baylor College of Medicine (BCM) and the National Institutes of Health (NIH) in the US.

Note that full information on the approval of the study protocol must also be provided in the manuscript.

Field-specific reporting

Please select the one below that is the best fit for your research. If you are not sure, read the appropriate sections before making your selection.

- [x] Life sciences
- [ ] Behavioural & social sciences
- [ ] Ecological, evolutionary & environmental sciences

For a reference copy of the document with all sections, see nature.com/documents/nr-reporting-summary-flat.pdf

Life sciences study design

All studies must disclose on these points even when the disclosure is negative.

Sample size	Our study focuses on an underrepresented clinical population where recruitment presents significant logistical challenges including geographic isolation, limited healthcare infrastructure, and historical underrepresentation in biomedical research. Given these constraints and the scientific imperative to diversify genomic studies beyond predominantly European-ancestry urban populations, we prioritized recruiting the maximum feasible sample size rather than targeting a predetermined number based on power calculations. Due to high prevalence of the specific phenotype in our study population, we expected the plausible variants may be enriched in our samples and the genetic variants may have larger effect size. The sample size of 711 children represents a substantial contribution to genomic diversity while providing sufficient power for meaningful genetic discovery in an underrepresented population, addressing critical gaps.
Data exclusions	We excluded individuals without a clear clinical diagnosis, individuals without adequate genotype data, or participants who were closely related to other individuals in the cohort.
Replication	Given the limitations and constraints of patient recruitment for this disorder, a replication cohort is not available; however, we sought and confirmed internal consistency between results from the two geographically disparate regions (central Africa and the Caribbean) under evaluation.
Randomization	Samples were randomized on the microarray chip (genotyping) and sequencing batches (flowcells) to reduce the potential for batch effect biases. To address potential population substructure that could lead to spurious associations, we included principal components (PCs) derived from genome-wide SNP data as covariates in all association analyses. In addition, we adjusted for key demographic variables (sex) that differed significantly between cases and controls.
Blinding	We specifically recruited individuals with SAM into the cohort based on their diagnostic category (ESAM or NESAM); there was no blinding in the recruitment process.

Reporting for specific materials, systems and methods

We require information from authors about some types of materials, experimental systems and methods used in many studies. Here, indicate whether each material, system or method listed is relevant to your study. If you are not sure if a list item applies to your research, read the appropriate section before selecting a response.

Materials & experimental systems

n/a	Involved in the study
☒	☐ Antibodies
☒	☐ Eukaryotic cell lines
☒	☐ Palaeontology and archaeology
☒	☐ Animals and other organisms
☐	☒ Clinical data
☒	☐ Dual use research of concern
☒	☐ Plants

Methods

n/a	Involved in the study
☒	☐ ChIP-seq
☒	☐ Flow cytometry
☒	☐ MRI-based neuroimaging

Clinical data

Policy information about clinical studies

All manuscripts should comply with the ICMJE guidelines for publication of clinical research and a completed CONSORT checklist must be included with all submissions.

Clinical trial registration

Not applicable.

Study protocol

The manuscript involves the use of demographic data and patient recruitment, but not for the purposes of clinical evaluation or intervention, and thus did not meet criteria for a clinical trial at the time of study enrollment. Inclusion and exclusion criteria for enrollment at both sites, and the encompassing protocol framework are given in PMID: 31857576.

Data collection

Collected clinical data included: diagnostic category (i.e. either ESAM or NESAM), basic anthropometry (e.g. height, weight), and clinical co-morbidities (e.g. fever, diarrhea) as outlined in PMID: 31857576. Genotype data was obtained from SNP microarray genotyping using the Infinium H3Africa Consortium Array v1.1; sequencing data was derived from short-read whole genome sequencing done on the Illumina Novaseq 6000.

Outcomes

not applicable

Plants

Seed stocks

Report on the source of all seed stocks or other plant material used. If applicable, state the seed stock centre and catalogue number. If plant specimens were collected from the field, describe the collection location, date and sampling procedures.

Novel plant genotypes

Describe the methods by which all novel plant genotypes were produced. This includes those generated by transgenic approaches, gene editing, chemical/radiation-based mutagenesis and hybridization. For transgenic lines, describe the transformation method, the number of independent lines analyzed and the generation upon which experiments were performed. For gene-edited lines, describe the editor used, the endogenous sequence targeted for editing, the targeting guide RNA sequence (if applicable) and how the editor was applied.

Authentication

Describe any authentication procedures for each seed stock used or novel genotype generated. Describe any experiments used to assess the effect of a mutation and, where applicable, how potential secondary effects (e.g. second site T-DNA insertions, mosiacism, off-target gene editing) were examined.
