# Supplementary material for: Aberrant one-carbon metabolism and ancestral genetics underlie edematous severe acute malnutrition
Source: Nat Commun. 2026 Oct 15;17:10798. doi: 10.1038/s41467-026-74239-x (PMC13669358; doi:10.1038/s41467-026-74239-x)
Supplement: Supplementary file 4 [file 41467_2026_74239_MOESM5_ESM.pdf]

nature portfolio

Peer Review File

Aberrant One-Carbon Metabolism and Ancestral Genetics Underlie Edematous Severe Acute Malnutrition

Corresponding Author: Dr Neil Hanchard

This file contains all reviewer reports in order by version, followed by all author rebuttals in order by version.

Version 0:

Reviewer comments:

Reviewer #1

(Remarks to the Author)
Context and Framing: The clinical problem being addressed in this manuscript is the differential mortality between patients with edematous and non-edematous malnutrition. There is a robust literature that informs current treatment guidelines for severe acute malnutrition that does take into account physiological and metabolic differences between the phenotypes, including impairments of one carbon metabolism. Clinical and public health care already takes place within a conceptualframework that specifically recognizes metabolic and physiologic brittleness as well as stenosed adaptation capacity in relation to metabolism of energy, protein, lipid, carbohydrate, limiting amino acids, and demand supply imbalances of mineral and vitamins. These cellular level impairments are conceptually placed at the pathophysiologic base of organ-level dysfunction including hepatic, renal, gastrointestinal and cardiorespiratory systems. The current therapeutic approach takes these into consideration.

Thus, while the magnitude of the contribution to one carbon metabolism of admixture variance through SNPs is of potential significance, its clinical significance and utility at the level of care guidelines is not clear.

The potential value of this work is in the view of this reviewer therefore, principally within the realm of genomics.

Within this realm, there are methodological issues which however might erode the confidence that can be placed on the findings and conclusions presented in this manuscript. These include:

Design Issues
The recruitment timeframes in Jamaica and Malawi very different, as are the age ranges of patients and the types of institutions providing patients (referral hospital versus rural hospitals). This heterogeneity of the sample populations might introduce bias.

Diagnostic criteria were different between sites Wellcome Criteria versus MUAC + edema. The overlap between the two is not complete and classification bias might have been introduced.

Cell free DNA is low quantity and fragmented. Failed genotypes for two of six SNPs suggest methods are fragile.

The use of this very small sample size of approximately 700 for 8m SNPs means the power to detect small differences is limited, raising the risk of both false negatives and spurious associations.

Whole genome sequencing was performed only on Malawian samples not on the Jamaican creating a bias towards East African genetics.

While these methodological problems do not invalidate the study they limit causal inference.

(Remarks on code availability)

Reviewer #2

(Remarks to the Author)
Lie et al. performed comprehensive genetic analyses on severe acute malnutrition (SAM) and both clinical subtypes of non-edematous SAM (NESAM) and edematous SAM (ESAM). The primary focus pertains to genetic association tests for specific one-carbon metabolism (OCM) associated loci. Lie et al. detected seven candidate loci associated with ESAM. By performing subsequent ancestral analyses, the authors reported that variants located in OCM-associated regions that are further associated with ESAM are mainly based on the East-African ancestry.

Major comments
• The authors need also to report the results of the whole GWAS analyses. Although the results will presumably not be genome-wide significant it is still urgent to report the results on the regions that were not shown here. The GWAS could be the secondary, explorative analysis. Data should be made available.
• Results should also be compared to GWAS data on thinness (e.g. PMID: 30677029) and on obesity (e.g. PMID: 30239722).
• Regarding the relevance of the leptin-melanocortin system on body weight regulation the authors should have a look on the genes within this system as well.
• As the birth weight has an effect on ESAM (line 677-679) the Barker hypothesis should also be discussed.
• A section regarding the limitations of the study needs to be included. Particularly regarding the lack of reference genomes for African populations and its implications as well as the Mendelian randomisation (limited number of SNPs, large 95% CI, etc.). MR with data of few individuals and SNPs may be underpowered.
• Please add a flow chart for all genetic analyses - which set of samples were used for which analyses (e.g., genotype-only). It is rather confusing following the various datasets referred to in the text. Same is true for the different terms for associated regions. Sometimes the authors refer to the associated SNPs as “ESAM-associated SNPs”, sometime they are called “OCM-ESAM SNPs”. Are these different entities? If not, please use one term throughout the whole manuscript and introduce the term in the flow chart.
• Did the authors check the sex assignment within the quality control steps for the genotyping data? Did the reported sex match the genetic sex? This can be checked in PLINK.
• Please add information on the phenotypes. How old were the individuals in each group? How many were female or male? What was the mean weight-for-age, etc.?
• Were the associated SNPs checked for associations with other diseases or trait? For instance, in the GWAS-catalogue?
• Page 16, Lines 472 ff.: The authors performed an MR on four ESAM-associated SNPs. Why were these SNPs selected? They are not stated in Supplementary Table 1 which includes all associated variants. Further, did the authors check pleiotropy for these SNPs?
Minor comments
• The authors should add the location and consequence of the SNPs being associated. Are the SNPs synonymous/non-synonymous? Or are they located within intronic regions?
• Page 14: iHS abbreviation was used before its introduction.
• Page 14, line 410: “… SNPs fell within 10kb…”. Previously (on Page 7, line 179) the authors state that a 50kb region was analysed. Here, they state 10kb. Was this a specific look-up or is this a typo?
• Page 19, line 561: “… including candidates MTHFR1, PRICKLE2 and PLD2, but only one OCM locus…”. Aren’t those three named candidates also OCM loci? Particularly as they are found to be associated within the association testing (see Supplementary Table 1, Figure 2) and those include OCM-associated regions checked for ESEM associations?
• Page 22, lines 644 ff.: Here, the authors mention heritability estimates, but these were included in the results sections. Are these calculated based on own data or is a reference missing at this point?
• Figure 6: please label the genes that surpass the threshold.
• Supplementary Table 1: are the position given those of the genes or already those regions included the 50kb adjacent region?

(Remarks on code availability)
Unfortunately, the URL does not work.

Reviewer #3

(Remarks to the Author)
I co-reviewed this manuscript with one of the reviewers who provided the listed reports. This is part of the Nature Communications initiative to facilitate training in peer review and to provide appropriate recognition for Early Career Researchers who co-review manuscripts.

(Remarks on code availability)
The provided URL does unfortunately lead to an empty github page.

Version 1:

Reviewer comments:

Reviewer #1

(Remarks to the Author)

Thank you for asking me to review this manuscript after the authors have taken on board reviewers' comments. I have reviewed the current version of the manuscript as well as the specific responses by the authors to questions raised by the reviewers. They (the authors) have thoughtfully and satisfactorily dealt with all the points raised. Conclusions indicated below flow from this review of the revised manuscript.

What are the noteworthy results? The targeting of genomic explorations on well-established metabolic findings that are important for pathophysiology of disease have yielded associations that reinforce the genetic findings put forward in the manuscript while avoiding some limitations of genetic epidemiology imposed by the observational nature of the project.

Will the work be of significance to the field and related fields? It should help to guide future investigations of genetic, epigenetic and other multi-omic approaches to elucidation of causation upon which clinical trials of new therapeutic approaches could more confidently rest.

Does the work support the conclusions and claims, or is additional evidence needed? The work supports the claims.

Are there any flaws in the data analysis, interpretation and conclusions? Do these prohibit publication or require revision?
None

Is the methodology sound? Does the work meet the expected standards in your field?
Is there enough detail provided in the methods for the work to be reproduced? Yes to all

Reviewer #2

(Remarks to the Author)
The authors answered all remarks elegantly. It would be great to also include the results of the additional analyses on GWAS data on thinness and obesity and on the leptin-melanocortin system in the ms.

Reviewer #3

(Remarks to the Author)
I co-reviewed this manuscript with one of the reviewers who provided the listed reports. This is part of the Nature Communications initiative to facilitate training in peer review and to provide appropriate recognition for Early Career Researchers who co-review manuscripts.

Open Access This Peer Review File is licensed under a Creative Commons Attribution 4.0 International License, which permits use, sharing, adaptation, distribution and reproduction in any medium or format, as long as you give appropriate

credit to the original author(s) and the source, provide a link to the Creative Commons license, and indicate if changes were made.
In cases where reviewers are anonymous, credit should be given to 'Anonymous Referee' and the source.
The images or other third party material in this Peer Review File are included in the article's Creative Commons license, unless indicated otherwise in a credit line to the material. If material is not included in the article's Creative Commons license and your intended use is not permitted by statutory regulation or exceeds the permitted use, you will need to obtain permission directly from the copyright holder.
To view a copy of this license, visit https://creativecommons.org/licenses/by/4.0/

Response to Reviewers: *Aberrant One-Carbon Metabolism and Ancestral Genetics Underlie Edematous Severe Acute Malnutrition.*

Reviewer #1 (Remarks to the Author):
Context and Framing: The clinical problem being addressed in this manuscript is the differential mortality between patients with edematous and non-edematous malnutrition. There is a robust literature that informs current treatment guidelines for severe acute malnutrition that does take into account physiological and metabolic differences between the phenotypes, including impairments of one carbon metabolism. Clinical and public health care already takes place within a conceptual framework that specifically recognizes metabolic and physiologic brittleness as well as stenosed adaptation capacity in relation to metabolism of energy, protein, lipid, carbohydrate, limiting amino acids, and demand supply imbalances of mineral and vitamins. These cellular level impairments are conceptually placed at the pathophysiologic base of organ-level dysfunction including hepatic, renal, gastrointestinal and cardiorespiratory systems. The current therapeutic approach takes these into consideration.

Thus, while the magnitude of the contribution to one carbon metabolism of admixture variance through SNPs is of potential significance, its clinical significance and utility at the level of care guidelines is not clear.

We thank the reviewer for their thoughtful consideration of our work, which, like most human genomic studies, is more geared towards providing unique insights to disease pathophysiology than to having immediate clinical implications. We consider the work to still be of added value, especially for the long-standing, under-represented, and under-managed disorder of edematous malnutrition. We have updated the manuscript (lines 134-136 and 659-661) to emphasize the potential of the research to inform future studies and to be a platform for consideration of therapeutic interventions and trials, without overpromising.

The potential value of this work is in the view of this reviewer therefore, principally within the realm of genomics.
Within this realm, there are methodological issues which however might erode the confidence that can be placed on the findings and conclusions presented in this manuscript. These include:

Design Issues
The recruitment timeframes in Jamaica and Malawi very different, as are the age ranges of patients and the types of institutions providing patients (referral hospital versus rural

hospitals). This heterogeneity of the sample populations might introduce bias.[misclassification]

Whilst we acknowledge that the cohorts are of differing ages and timeframes, our study is predicated on the inherent advantages of studying germline genetic variation - that is, stable inherited DNA sequence that is unchanged by disease status, age, institution, or recruitment. We operate on the premise that in a case-control context, as long as the primary clinical designations of cases and controls are consistent, the effect of other parameters that could confound more labile biomarkers is mitigated. This framework has facilitated statistically robust, large scale, human genomic studies of common complex traits such as diabetes and obesity using individuals recruited at different ages, stages, and locations. This approach also facilitates a broader generalizability of the findings.

Diagnostic criteria were different between sites Wellcome Criteria versus MUAC + edema. The overlap between the two is not complete and classification bias might have been introduced.

We thank the reviewer for this observation. To clarify, starting in our study of DNA methylation in these cohorts, we were made aware of the importance of congruence between diagnostic criteria. We thus reclassified individuals from Malawi (where we had both MUAC and weight-for-age) to the Wellcome Criteria. The vast majority of individuals did not change classification (+/- edema is independent of MUAC), and the few individuals that were reclassified as moderate- instead of severe- malnutrition were removed from the analysis. We have clarified this procedure on lines 169-173 of the revised manuscript.

Cell free DNA is low quantity and fragmented. Failed genotypes for two of six SNPs suggest methods are fragile.

We initially included the failed SNPs to maintain methodological transparency with regard to the availability and usability of cfDNA from small volume serum samples; we have maintained this in the supplementary methods, but have removed those SNPs from **Supplementary Table S2** to improve clarity and focus. It is true that cfDNA is more fragmented than nascent extracted DNA; it is also present in smaller quantities, at least with current extraction techniques. This observation drove our choice to employ targeted- rather than genome-wide genotyping; this allowed us to manually inspect individual genotypes closely and remove assays and individuals that did not meet quality control. We consider the congruency of minor allele frequencies between cDNA and SNParray genotyped SNPs to be a testament to the fidelity of this approach (revised **Supplementary Table S2**)

The use of this very small sample size of approximately 700 for 8m SNPs means the power to detect small differences is limited, raising the risk of both false negatives and spurious associations.

Sample size is always a consideration in these types of studies. We note, however, that our primary association analyses were focused on the subset of 45,411 imputed- and 9,479 genotyped SNPs at OCM loci (lines 414-416 in the original manuscript and now clarified in updated **Supplementary Figure 1A**) . Most of these SNPs are in linkage disequilibrium with nearby SNPs and thus not independent of each other; as result, our Bonferroni corrected thresholds for locus and SNP level significance are likely conservative for this dataset. We also decided to limit our top loci to those with multiple SNPs surpassing our threshold, removing a strong single SNP association in *MTHFR* that did not change any of the downstream conclusions or results. A threshold for significance based on the number of SNPs genotyped has been robust for genetic association studies, with few reported spurious associations, and our downstream validation in cfDNA is consistent with this. We agree that the power to detect small differences (e.g., odds ratios < 1.5) is limited and, as with any genetic association study, there may be false negatives; however, the larger effects noted at the significant OCM loci or in the cumulative association are within the scope of the study and it is these 'major' effects that were of more interest to us. In the revised discussion (lines 656-659) we look forward to forthcoming ESAM genome-wide studies with larger sample sizes that can evaluate loci with smaller effect sizes.

Whole genome sequencing was performed only on Malawian samples not on the Jamaican creating a bias towards East African genetics.

To clarify, WGS data was only used to look at/for signatures of recent selection (lines 376-378) and was not a part of the association analyses. We found that the primary association results from genome-wide genotyping held true across both populations (lines 503-504; Suppl. Table S5). The WGS-derived selection signals in Malawi were also contextualized alongside similar WGS-signals in seven African populations from across the continent (lines 394-397 in updated manuscript). Recent continental admixture can result in long tracks of linkage disequilibrium, confounding iHS selection signals. Therefore, we did not calculate selection in the Afro-European admixed population of Jamaica; however, the African populations assessed include putative parental ancestries for Jamaica that are relevant to the ESAM phenotype. We have clarified this analytical plan in a newly conceived **Figure 1B** in the revised manuscript which provides an overview of the datasets used and analyses performed.

While these methodological problems do not invalidate the study they limit causal inference.

We agree and were careful not to invoke causality surreptitiously. We have updated the discussion (lines 618-620) to emphasize that while these SNPs could partially mediate the metabolic differences between ESAM and NESAM, it would be incorrect to invoke independent causality from the data presented.

Reviewer #2 (Remarks to the Author):

Lie et al. performed comprehensive genetic analyses on severe acute malnutrition (SAM) and both clinical subtypes of non-edematous SAM (NESAM) and edematous SAM (ESAM). The primary focus pertains to genetic association tests for specific one-carbon metabolism (OCM) associated loci. Lie et al. detected seven candidate loci associated with ESAM. By performing subsequent ancestral analyses, the authors reported that variants located in OCM-associated regions that are further associated with ESAM are mainly based on the East-African ancestry.

Major comments

• The authors need also to report the results of the whole GWAS analyses. Although the results will presumably not be genome-wide significant it is still urgent to report the results on the regions that were not shown here. The GWAS could be the secondary, explorative analysis. Data should be made available.

As anticipated, none of the genome-wide association results met genome-wide significance - the resulting Manhattan and QQplot have been included as **Supplementary Figure S11** and referenced in lines 654-655. We have also included the summary statistics for SNPs with $p<10^{-4}$) in **Supplementary Table S8**. We agree that these sites and loci could be a useful lookup for related traits; however, we suspect that our current, ongoing GWAS efforts as part of the Kwashiorkor Study Network (KwashNet - https://www.genome.gov/research-at-nhgri/projects/KwashNet) will be even more informative. We have updated lines 655-658 of the updated manuscript with this data.

• Results should also be compared to GWAS data on thinness (e.g. PMID: 30677029) and on obesity (e.g. PMID: 30239722).

We thank the reviewer for such an interesting and intriguing idea. We have previously noted the overlap between epigenomic signatures found in other general anthropometric parameters and in SAM. In line with this suggestion, we evaluated our SAM association

statistics for the top (genome-wide significant) variants from each of the GWAS noted; however, we did not find evidence of co-association in our dataset. Some of this reflects sparsity in SNP overlap at the quriered loci, especially with the substantial differences in population/ancestry and sample size. For instance, from the large meta-analysis of body fat distribution including 694,649 individuals of European ancestry, 346 genetic variants were associated with waist-to-hip ratio. Among these, one of the top variants, located on chromosome 17 (chr17:17,493,272; rs4646342) is present in our OCM-50K region dataset, but the variant did not show evidence of association in our analysis (β = 0.013, Wald p = 0.62). In addition, for associations on chromosomes 2 and 21, there were 10 variants in the OCM-50K region dataset within ±500 base pairs of the top variants reported, but none of these variants reached statistical significance in our study. Lastly, we evaluated 19 variants identified as top hit in comparisons of obese versus thin and obese versus control individuals in *"Genetic architecture of human thinness compared to severe obesity,"* ; unfortunately, none of these variants were present within ±500 base pairs of variants in our OCM-50K region dataset. These results suggest that OCM loci may not have the same effect on anthropology that they do on ESAM, although we cannot discount that there are other loci overlapping both phenotypes in appropriately powered GWAS of ESAM, and anticipate more robust colocalization analyses from such studies (see KwashNet above).

• Regarding the relevance of the leptin-melanocortin system on body weight regulation the authors should have a look on the genes within this system as well.

We looked at the summary statistics for SNPs within and proximal to genes annotated to the Leptin-Melanocortin pathways, but did not find strong evidence of association (e.g. at a suggestive GWAS threshold), with the same caveats as above.

• As the birth weight has an effect on ESAM (line 677-679) the Barker hypothesis should also be discussed.

We have expanded upon the potential relevance of the Barker hypothesis and genetic pleiotropy in SAM outcomes in lines 687-691, including relevant citations/publications.

• A section regarding the limitations of the study needs to be included. Particularly regarding the lack of reference genomes for African populations and its implications as well as the Mendelian randomization (limited number of SNPs, large 95% CI, etc.). MR with data of few individuals and SNPs may be underpowered.

We thank the reviewer for this suggestion, and have crafted a separate paragraph outlining potential limitations including sample size, reference genomes, and LD coverage in the ancestral populations evaluated, and these are included in lines 703-715 of the revised manuscript.

"Our results provide a novel view of the SAM-NESAM dichotomy; however, there are limitations to our study that should be noted. Aside from the sample size limitations already noted, we were limited in our coverage of genomic variation. The H3Africa SNP array offers a robust coverage of LD among genetically African ancestries; however, it is far from fully comprehensive. Although genome sequencing efforts in Africa (and among populations of predominant African ancestry) are increasing; the number of reference genomes, particularly for disparate African groups, is still much smaller than that available for European ancestry groups. As a result, accurate and confident imputation, and parental ancestry mapping were particularly challenging in our study, and may have led to false negative or overly conservative results. This highlights the need for larger and more extensive genetic databases of sequence variation from a broad cross-section of African populations, particularly from foundational groups with unique ancestries. Subsequent integration and curation of the derived data into reference panels and widely accessible databases of African genetic variation will facilitate more robust studies. "

• Please add a flow chart for all genetic analyses - which set of samples were used for which analyses (e.g., genotype-only). It is rather confusing following the various datasets referred to in the text. Same is true for the different terms for associated regions. Sometimes the authors refer to the associated SNPs as "ESAM-associated SNPs", sometime they are called "OCM-ESAM SNPs". Are these different entities? If not, please use one term throughout the whole manuscript and introduce the term in the flow chart.

We thank the reviewer for prompting this clarification. We have created a "Visual abstract" type figure (Figure 1B in the revised manuscript) that provides a clearer delineation of the datasets used and analyses performed; this includes a consistent terminology for SNPs at OCM loci that are associated with ESAM ("ESAM-associated OCM SNPs"). This is meant to complement the more detailed analysis flowchart found in **Supplementary Figure S1**.

• Did the authors check the sex assignment within the quality control steps for the genotyping data? Did the reported sex match the genetic sex? This can be checked in PLINK.

Yes as part of our QC check, sex-gender discrepancies were noted and evaluated; those that could not be resolved (e.g. as a data entry error) were flagged for removal. This is now reflected on lines 202-204 in our updated flowchart (**Supplementary Figure S1)**.

• Please add information on the phenotypes. How old were the individuals in each group? How many were female or male? What was the mean weight-for-age, etc.?

We have updated this data in **Supplementary Table S3**, to include the relative proportions of males (58.5% in Jamaica; 55% in Malawi), and age (mean 16.9 months in Jamaica; 17.2 in Malawi).

• Were the associated SNPs checked for associations with other diseases or trait? For instance, in the GWAS-catalogue?

Yes, we checked for association in the GWAS catalogue using the rsIDs of the top SNPs. Only one SNP (rs11130959) has also been associated with another phenotype – Brain shape, which likely represents the known neurodevelopmental effects of the *PRICKLE2* gene. We cannot discount that surrounding SNPs at the same loci might be associated with other phenotypes.

• Page 16, Lines 472 ff.: The authors performed an MR on four ESAM-associated SNPs. Why were these SNPs selected? They are not stated in Supplementary Table 1 which includes all associated variants. Further, did the authors check pleiotropy for these SNPs?

We thank the reviewer for requesting more clarity here as it pointed us to an apparent copy-paste error in the former Supplementary Table S4. We have now updated Table S4 to have the correct SNP IDs and have highlighted the SNPs that were genotyped in the serum cell free DNA samples. We have also clarified in the methods that these SNPs were selected to be the SNPs with the lowest P values at the OCM loci **that also had** favorable surrounding sequence for PCR amplification. In one case this meant utilizing a proxy SNP in strong linkage disequilibrium (D'=0.89). We have highlighted the overlapping SNPs in **Supplementary Table S4**, and updated the **Supplementary Methods** accordingly.

We did check for pleiotropy among these SNPs, but did not find evidence supporting this.

Minor comments

• The authors should add the location and consequence of the SNPs being associated. Are the SNPs synonymous/non-synonymous? Or are they located within intronic regions?

We have added an additional column to **Supplementary Table S4** with the SNP annotation in addition to stating their functional consequence in the results (e.g. line 439 in the revised manuscript as well as lines 431, and 444).

• Page 14: iHS abbreviation was used before its introduction.

We have changed and updated this accordingly.

• Page 14, line 410: "... SNPs fell within 10kb...". Previously (on Page 7, line 179) the authors state that a 50kb region was analysed. Here, they state 10kb. Was this a specific look-up or is this a typo?

We thank the reviewer for pointing this out; this is indeed a typo that has been clarified in the revised version.

• Page 19, line 561: "... including candidates MTHFR1, PRICKLE2 and PLD2, but only one OCM locus...". Aren't those three named candidates also OCM loci? Particularly as they are found to be associated within the association testing (see Supplementary Table 1, Figure 2) and those include OCM-associated regions checked for ESEM associations?

This was a grammatical faux pas in our attempt to contrast the local ancestry-limited results. The statement should read "*....when conditioned on shared East African ancestry (proxied by LWK), seven loci surpassed the loci-level threshold for association, including candidates MTHFR1, PRICKLE2, and PLD2**; however,** only one OCM locus and none of our candidates, met the same significance criterion when adjusting for West African ancestry...*" and has been corrected as such (now line 563).

• Page 22, lines 644 ff.: Here, the authors mention heritability estimates, but these were included in the results sections. Are these calculated based on own data or is a reference missing at this point?

We performed a preliminary assessment of heritability from the genome-wide data; however, with the small sample size for this purpose, we did not want these results to be a primary focus, and so included them as a discussion point. We have updated the prose to indicate the methods and parameters used (lines 647-648).

• Figure 6: please label the genes that surpass the threshold.
We have updated the figure to include all of the top loci of interest.

• Supplementary Table 1: are the position given those of the genes or already those regions included the 50kb adjacent region?
The positions in Suppl. Table 1 include 50kb upstream of the coding gene start and 50 kb downstream of the last exon. We have clarified this in the Table legend.

Reviewer #2 (Remarks on code availability):

- Unfortunately, the URL does not work.

Our apologies for the inconvenience; the link is now fixed and should be working.

Reviewer #3 (Remarks to the Author):
I co-reviewed this manuscript with one of the reviewers who provided the listed reports. This is part of the Nature Communications initiative to facilitate training in peer review and to provide appropriate recognition for Early Career Researchers who co-review manuscripts.

Reviewer #3 (Remarks on code availability):
The provided URL does unfortunately lead to an empty github page.
Thank you for time in helping to provide an insightful and thoughtful review. As noted above, the link is now fixed and should be working.

Response to Reviewers:

Reviewer #2 (Remarks to the Author):
It would be great to also include the results of the additional analyses on GWAS data on thinness and obesity and on the leptin-melanocortin system in the ms.

We are pleased that the reviewer found our responses appropriate and felt the manuscript was now appropriate for publication. In response to the request to include the thinness and obesity analyses, we have updated the discussion (lns 396-406) to read:

Our previous epigenomics study noted overlap between ESAM differentially methylated genes and genes associated with general anthropometric parameters. We thus sought to evaluate our OCM SNP regions for co-association between ESAM and body anthropometry; however, only ten of the 364 genome-wide significant variants associated with waist-to-hip ratio (n=346 variants)[REF], and none of the 19 genome-wide significant variants associated with body size (obese vs thin individuals) [REF], overlapped our OCM regions, and none of the overlapping SNPs showed evidence of association with ESAM. It may be that OCM loci do not have the same direct effect on anthropometry that they do on ESAM, although we cannot discount that an appropriately powered GWAS of ESAM could still identify co-localizing SNPs that impact both phenotypes. Genome-wide studies of ESAM (and related phenotypes) would be well served by the development of larger cohorts, which we have spearheaded through the establishment of the Kwashiorkor Study Network (KwashNet – see Data Availability).
